# NucHMM: a method for quantitative modeling of nucleosome organization identifying functional nucleosome states distinctly associated with splicing potentiality

**DOI:** 10.1186/s13059-021-02465-1

**Published:** 2021-08-26

**Authors:** Kun Fang, Tianbao Li, Yufei Huang, Victor X. Jin

**Affiliations:** 1 Department of Molecular Medicine, UTHSA-UTSA Joint Biomedical Engineering Program, San Antonio, TX 78229 USA; 2 Department of Molecular Medicine, University of Texas Health Science Center at San Antonio, San Antonio, TX 78229 USA; 3 Department of Medicine, UPMC Hillman Cancer Center, University of Pittsburgh, Pittsburgh, PA 15232 USA

**Keywords:** Nucleosome organization, Hidden Markov model, Splicing potentiality

## Abstract

We develop a novel computational method, NucHMM, to identify functional nucleosome states associated with cell type-specific combinatorial histone marks and nucleosome organization features such as phasing, spacing and positioning. We test it on publicly available MNase-seq and ChIP-seq data in MCF7, H1, and IMR90 cells and identify 11 distinct functional nucleosome states. We demonstrate these nucleosome states are distinctly associated with the splicing potentiality of skipping exons. This advances our understanding of the chromatin function at the nucleosome level and offers insights into the interplay between nucleosome organization and splicing processes.

**Supplementary Information:**

The online version contains supplementary material available at 10.1186/s13059-021-02465-1.

## Background

A nucleosome is the fundamental structural unit of eukaryotic chromatin and nucleosome core is formed by the wrapping of 146-bp DNA in 1.75 left-handed superhelices around a histone octamer [1–3]. Nucleosome organization, described as nucleosomal phasing, spacing, and positioning, is determined by the interplay among nucleosome, nucleosome-binding factors such as DNA-binding factors, histone chaperones, and ATP-dependent chromatin remodelers [4, 5]. Several models, supported by substantial experimental findings, have been proposed for determining nucleosome organization: (1) DNA-binding factors or ATP-dependent chromatin remodelers forcing nucleosome depletion in certain genomic regions [6–8]; (2) the intrinsic DNA sequence patterns preferring histone binding [9–11]; and (3) a barrier statistically favoring deposition of a well-positioned nucleosome and forcing the periodic positioning of all other nucleosomes [12]. Despite of these elegant models, there still lacks a quantitative model to determine the combinational effects of the different influencing factors on nucleosome organization. For example, can nucleosome organization be quantitatively classified into distinct nucleosome states? How many nucleosome states are there in an epigenome? How many characteristic features are there in a particular nucleosome state? What are the relationships among these features? Are nucleosome states cell type-specific and/or genomic regional-specific?

Many studies have revealed that nucleosome organization plays a key role in the regulation of gene expression [4, 5, 13–15]. Genome-wide nucleosome mapping has also provided structural and mechanistic links among nucleosome, wrapped DNA, and nucleosome-binding factors [16–18] and elucidated novel functionalities of organized nucleosomal arrays in an unbiased way [19–21]. Recent studies have found that chromatin structure, in terms of nucleosome organization and specific histone modifications, acts as key regulators of alternative splicing. These studies provided evidence that there exists crosstalk between chromatin and splicing [22–24]. Among these studies, genome-wide mapping of nucleosomes has clearly illustrated the enrichment of nucleosomes at intron-exon junctions [25–27]. Other works, including ours, has revealed a strong correlation between several histone modifications across the alternatively spliced regions and splicing outcome [28, 29]. However, these findings are mostly correlative and observational. Therefore, it is imperative to develop a computational model to examine their relationship quantitatively.

Although many computational methods were developed to determine epigenetic states [30–60], several limitations include that (1) some supervised learning methods such as ChromaSig [60] cannot find de novo information, and (2) some unsupervised learning methods such as HMMSeg [31], ChomHMM [35], Segway [39], and T-cep [59] cannot optimally capture spatial patterns of the epigenetic marks on the nucleosomes, and they were not designed with modeling nucleosome organization. Thus, none of the above methods can define functional nucleosome states, i.e., states encoding combinatorial histone marks and nucleosome organization features that perform specific functions and respond to the different environment and intercellular signaling. Our knowledge at the quantitative aspect is very limited about the phasing of a nucleosome array, the spacing between two dyads of the nucleosomes, the degree of nucleosome positioning, as well as the extent to which the combinatorial epigenetic pattern influences nucleosome organization. There is a lack of quantitative measures on the association of functional nucleosome states with the splicing potentiality of skipping exons (SEs).

In this study, we develop a novel computational method, NucHMM, which integrates a hidden Markov model (HMM) with the characteristics of nucleosome organization (phasing, spacing, positioning), to identify the nucleosome states associated with cell type-specific combinatorial histone marks. We test it on publicly available MNase-seq and ChIP-seq of H3K4me1, H3K4me3, H3K27ac, H3K36me3, H3K79me2, H3K9me3, and H3K27me3 data in MCF7, H1, and IMR90 cells [61] and identify cell type-specific functional nucleosome states. We further quantitatively measure the association of functional nucleosome states with the splicing potentiality of SEs. Our work advances our understanding of chromatin function at the nucleosome level and further offers mechanistic insight into the interplay between nucleosome organization and splicing process.

## Results

### An overview of NucHMM

**Fig. 1:**
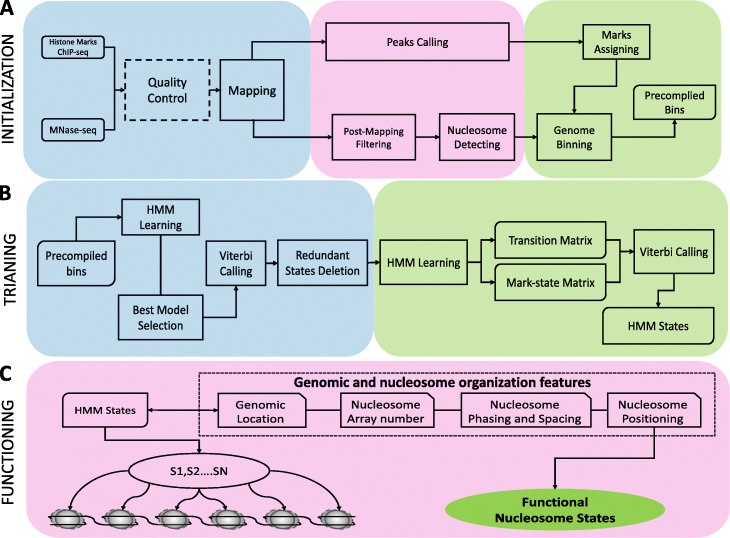
An overview of NucHMM workflow. **A** The initialization step combines several existing tools to construct nucleosome-level HMM training sequences. **B** The training step includes a selection of the best model and two rounds of HMM training for the best selected model. BW algorithm is applied to acquire the transition probability matrix and the mark-state matrix derived from the emission probability matrix. **C** The functioning step performs functional screening on the nucleosomes based upon the genomic location, nucleosome array number, nucleosome spacing, phasing, and positioning and finally identifies the functional nucleosome states

To quantitatively modeling the nucleosome organization, we have developed a novel algorithm, NucHMM, to identify functional nucleosome states. NucHMM is composed of three consecutive modules: (1) initialization, (2) training, and (3) functioning (Fig. 1 and the “Methods” section). Briefly, the initialization module pre-processes the raw sequencing data into the readable data input for the training module including converting fastaq into bam, calling the peaks for ChIP-seq data by MACS2 [62] or EPIC2 [63], identifying the positioned nucleosomes from MNase-seq by iNPS [64], and binning the genome based on positioned nucleosomes where each nucleosome-bin is assigned with an observation symbol from an alphabet list of 2^*n*^ observations symbols representing each possible combination of the number (*n*) of histone marks. The training module is composed of two rounds of HMM training. The first round is to train multiple HMMs for 300 iterations and to select the best HMM based on the smallest BIC score. The second round is to retrain the best HMM for another 200 iterations (Additional file 1: Fig. S1) after revising the input as aborting the states with very few bins (lower than 0.5% of the total nucleosomes or a user-defined cutoff) and evenly redistributing the transition probabilities of the aborted states to the remaining states. The resulting HMM further uses the Viterbi decoding algorithm to obtain the HMM states at the nucleosome level. The functioning module defines the functional nucleosome states (NucSs) by associating each of HMM states with genomic and nucleosome organization features, including (1) genomic location—identifying the most enriched genomic regions for each of HMM states; (2) an average number (Ave No.) of nucleosomes—identifying an average of the number of nucleosomes in a nucleosome array for each of HMM states, where a nucleosome array is defined as a set of nucleosomes that have the same state and the distance between two adjacent nucleosomes is less than 350 bp (Additional file 1: Fig. S2); (3) nucleosome phasing and spacing—determining nucleosome phasing score and average (Ave) spacing for each of HMM states by filtering out those nucleosomes if their spacing is out of a defined range (see the “Methods” section—Eq. 6); and (4) nucleosome positioning—determining nucleosome positioning score for each of HMM states by firstly building the group containing all nucleosome positioning scores for each state and then filtering out the nucleosomes with user-defined nucleosome positioning cutoff (by default, NucHMM will use 0.05 and 0.95 quantile values of the group as the cutoff).

### Selecting the best HMM and determining the genomic location

**Fig. 2:**
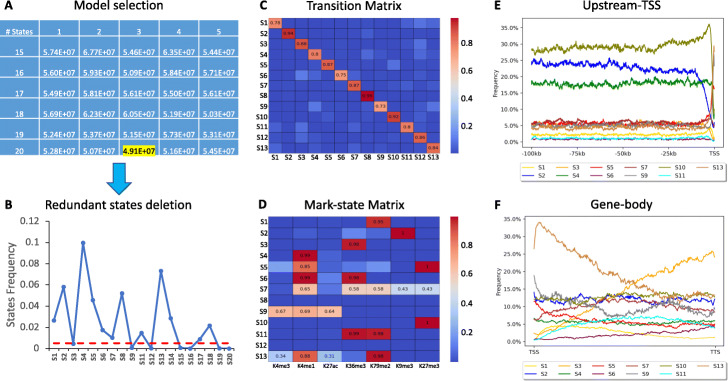
Selecting the best HMM and defining genomic regions for each of HMM states. **A** We trained 50 HMM models (other 25 models were shown in Additional file 1: Table S4) with different numbers of initial states and select the best model with the smallest BIC score (the highlighted model). **B** A line plot showed seven states are redundant in the current “best” model. We applied the second round HMM training by removing those seven redundant states. **C** The transition probabilities of the final 13-states HMM. The transitions were from states on *y*-axis to the *x*-axis. **D** The mark-state probabilities that derived from the emission probabilities. Each column represents a histone mark and each row represents a HMM state. **E** A distribution of each of HMM states in 100Kb Upstream TSS. **F** A distribution of each of HMM states in the gene body

We tested NucHMM in publicly available MNase-seq and ChIP-seq of H3K4me1, H3K4me3, H3K27ac, H3K9me3, H3K27me3, H3K36me3, H3K79me2 data in MCF7, H1, and IMR90 cell types (Additional file 1: Tables S1-2). We used iNPS to identify 11.6, 11.9, and 12.7 million genome-wide positioned nucleosomes in MCF7, H1, and IMR90 cell types, respectively. Since the functional nucleosomes are likely located in close to 5′transcription start site (5TSS), we chose a gene-centric genomic region for training HMM ranging from − 100Kb upstream to 5TSS (Upstream-TSS), gene body (Gene-body), and + 10Kb downstream of transcription terminal site (TTS) (Downstream-TTS) (Additional file 1: Suppl. Notes). Thus, only around 7.2, 7.4, and 7.3 million positioned nucleosomes for MCF7, H1, and IMR90 cell types were used for the first round training. We trained a total of 50 HMMs with five initial states ranging from 15 to 25 each repeated by five times and selected the best model with 20 initial states based on its smallest BIC score, 4.91E+07 (Fig. 2A and Additional file 1: Tables S3-5). We found seven states in the best model that are redundant (Fig. 2B) and thus removed them before the second round of training (Additional file 1: Suppl. Notes and Fig. S3). We finally achieved a model with 13 HMM states with a transition matrix showing the transition probabilities among states (Fig. 2C) and a mark-state matrix showing the emission probabilities for each of the seven marks in each of the 13 HMM states (Additional file 1: Fig. 2D and Fig. S4). We compared our HMM states to ChromHMM/Segway states and confirmed that our HMM is capable of capturing the chromatin states with the improved nucleosome level (Additional file 1: Suppl. Notes and Figs. S5-8). We further performed genomic location analysis and observed state 2 with H3K9me3 mark, state 4 with H3K4me1, and state 10 with H3K27me3 mark were highly enriched in the Upstream-TSS, particularly in Distal/Proximal (− 100Kb to − 1Kb upstream to 5TSS), and state 5 with H3K27me3/K4me1 marks, state 7 with H3K4me1/K36me3/K79me2/K9me3/K27me3 marks, and state 9 with H3K4me1/K4me3/K27ac marks were modestly enriched in the same region (Fig. 2E). As expected, states with H3K36me3 or H3K79me2 marks including state 1 with H3K79me2, state 3 with H3K36me3, state 6 with H3K4me1/K36me3 marks, state 11 with H3K36me3/K79me2 marks, and state 13 with H3Kme1/K4me3/K27ac/K79me2 were highly enriched in the Gene-body and Downstream-TTS (Fig. 2F and Additional file 1: Figs. S9-11). States 8 and 12 were not enriched with any known marks, thus not included for further functional characterization.

### Determining nucleosome phasing and spacing

**Fig. 3:**
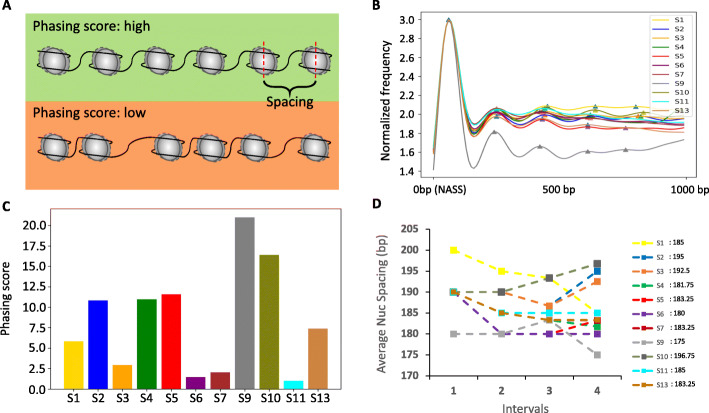
Nucleosome phasing and spacing of each of HMM states. **A** A schematic diagram shows the definition of nucleosome phasing and spacing. The upper nucleosome array has a higher phasing score than the bottom nucleosome array does. Nucleosome spacing is the distance between two dyads of the nucleosomes. **B** A nuc-array-coverage plot showed the normalized nucleosome array frequency coverage of each state in the 1 kb range. 0 is the start position of the array with a certain HMM state. **C** A bar plot showed the phasing score of each HMM state calculated by Welch’s method. **D** A line plot of nucleosome spacing. The interval is the distance between two peaks in panel B which is also the nucleosome spacing. We averaged 4 nucleosome spacing in the 1Kb nucleosome array as the nucleosome spacing value. The final nucleosome spacing values for each state were shown next to the legend

Nucleosome phasing and spacing are two main features to characterize nucleosome organization (Fig. 3A). We mathematically defined the nucleosome phasing score and spacing value based on the distribution signals of the nucleosome arrays (see the “Methods” section). We first plotted nucleosome array frequency and clearly observed distinct coverage patterns associated with each of HMM states (Fig. 3B). We then calculated the phasing score for each of HMM states by Welch’s method and found that states 5, 9, and 10 have the highest phasing score (Fig. 3C and Additional file 1: Fig. S12), suggesting that H3K4me1 and H3K27me3 marks may be capable of imposing a better organized nucleosome array. We then derived the average of nucleosome spacing for each of HMM states after averaging four nucleosome spacing values within the 1Kb nucleosome array (Fig. 3D). Interestingly, we found states 2, 3, and 10 with two repressive marks H3K9me3 and H3K27me3 and one elongation mark H3K36me3 tend to have larger nucleosome spacing values, while states 5, 6, and 9 associated with active marks H3K4me1 and H3K27ac have smaller spacing values. We further verified the reliability of our methods for calculating the phasing score and spacing value by using a simulated nucleosome array coverage signal (Additional file 1: Suppl. Notes and Fig. S13). Taken together, our results strongly suggest nucleosome phasing and spacing are intimately correlated with distinct functionality of different histone marks.

### Determining nucleosome positioning and defining functional nucleosome states

**Fig. 4:**
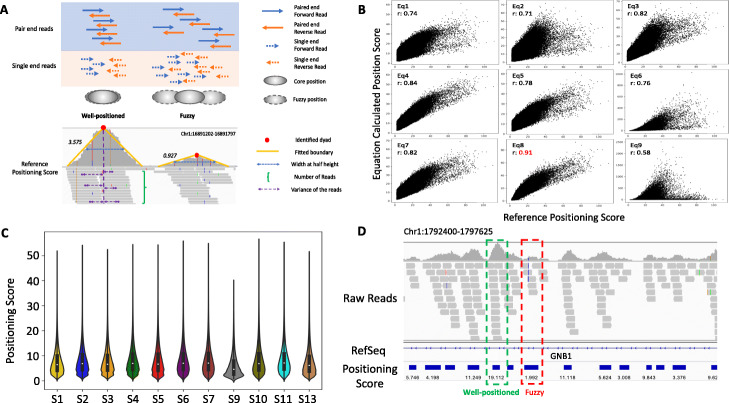
Nucleosome positioning of each of HMM states. **A** The illustration of nucleosome positioning definition and reference positioning score calculation. The upper panel showed the definition of well-positioned as well as fuzzy nucleosome. The bottom panel schematically drew the elements that were used to calculate the reference nucleosome positioning: width at half height restricted by fitted boundary, the number of reads and the variance of the reads centered by the identified dyad. **B** The correlation between each iNPS-derived positioning equation and reference positioning score equation in MCF7 cell type. Equation 8 showed the best correlation with the reference positioning score. **C** The violin plots showed the distribution of the nucleosome positioning score of each HMM states. **D** IGV visualized the nucleosome reads distribution and the positioning score calculated by Eq. 8. Green box shows the well-positioned nucleosome with a high positioning score, and red box shows the fuzzy nucleosome with a low positioning score

Nucleosome positioning is the most important characteristic of nucleosome organization and is often qualitatively classified as well-positioned or fuzzy (Fig. 4A—upper). We first defined a reference nucleosome positioning (rNP) score based on the pile-up of raw reads (see the “Methods” section—Eq. 7 and Fig. 4A—lower). We then tested nine empirical equations (Additional file 1: Suppl. Notes) on the positioned nucleosomes by the Pearson correlation with rNP to derive a final equation to determine the NP score or the degree of nucleosome positioning (see the “Methods” section—Eq. 8 and Fig. 4B, Additional file 1: Fig. S14). We found the distributions of the nucleosome positioning scores showed a slightly difference among HMM states (Fig. 4C) and defined the mean of each distribution as the nucleosome positioning score. An IGV visualization of a genomic region for the nucleosome reads distribution and the positioning score calculated by Eq. 8 was shown in Fig. 4D.

After examining the HMM states with four genomic regions and nucleosome organization features, we defined 11 functional nucleosome states (NucSs) (Table 1) with a detailed description and visualization of each of 11 NucSs in Additional file 1: Suppl. Notes and Fig. S15. Cell type-specific NucSs-genes analysis and the lists were found in Additional file 1: Suppl. Notes, Fig. S16 and Additional files 2, 3 and 4, respectively.

**Table 1:** The definition of functional nucleosome states

Functional nucleosome states (HMM states)	Histone marks	Ave. no. of nucleosomes	Genomic location	Ave. spacing	Positioning score	Phasing score
Elongation accelerator NucS1 (S1)	K79me2	3.99	5′-gene body	185.00	7.97	5.84
Compacting organizer NucS2 (S2)	K9me3	8.49	Distal	195.00	8.48	10.82
Elongation stabilizer NucS3 (S3)	K36me3	5.82	3′-gene body	192.50	8.06	2.93
Accessible booster NucS4 (S4)	K4me1	4.13	Distal	181.75	8.47	10.96
Primed intermediator NucS5 (S5)	K4me1/K27me3	4.56	Distal/promoter	183.25	8.75	11.58
Elongation terminator NucS6 (S6)	K4me1/K36me3	3.74	3′-gene body	180.00	8.61	1.46
Elongation processor NucS7 (S7)	K4me1/K36me3/K79me2/K9me3/K27me3	5.32	Gene body	183.25	8.90	2.04
Transcriptional stimulator NucS8 (S9)	K4me3/K4me1/K27ac	2.09	Promoter	175.00	6.07	21
Crowding controller NucS9 (S10)	K27me3	7.37	Distal	196.75	8.82	16.4
Elongation speeder NucS10 (S11)	K36me3/K79me2	4.55	Gene body	185.00	8.72	1
Elongation initiator NucS11 (S13)	K4me3/K4me1/K27ac/K79me2	4.67	Promoter/5′-gene body	183.25	7.81	7.39

### Determining the splicing potentiality of SEs

**Fig. 5:**
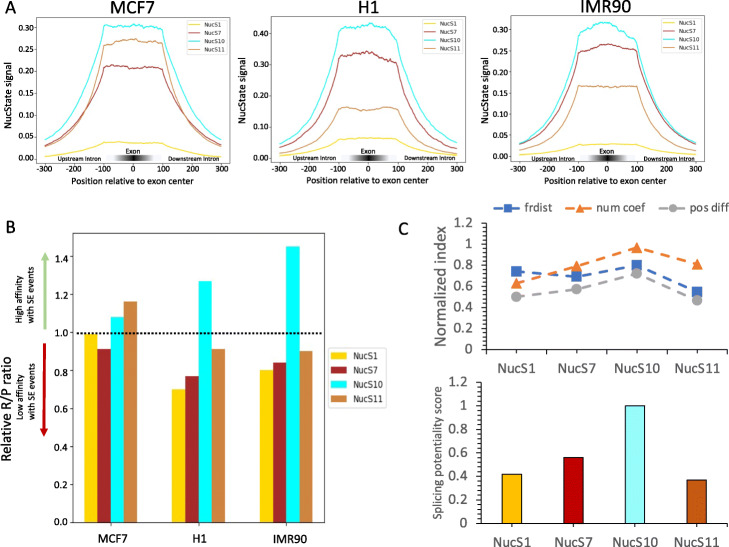
The functional nucleosome states associated with the splicing potentiality of SEs. **A** H3K79me2-related NucSs signals center on exons. NucS10 showed the highest enrichment in exons. **B** A bar plot showed different SE affinity of each H3K79me2-related NucSs in each cell type. Briefly, we counted the raw number of SE events for each H3K79me2-related NucSs in each cell type and then assumed SE event was randomly associated with NucSs to get the predicted SE events. Finally, we used the ratio of raw vs predicted number as the SE event affinity for each H3K79me2-related NucS. NucS10 showed a higher SE affinity among all three cell types. **C** Semi-quantifying the SE potentiality of each H3K79me2-related NucSs. The NucS10 consistently had the highest SE potentiality score. We assessed SE potentiality from three aspects: the Frechet distance between the nucleosome distribution of SE and no-SE; the nucleosome positioning difference between nucleosomes in SE and no-SE region; the normalized SE events of each NucS

H3K79me2 mark has been reported to be functionally associated with elongation and splicing processes [28, 29]; we were thus particularly interested in understanding the functional relationship of SEs with NucS1 (elongation accelerator), NucS7 (elongation processor), NucS10 (elongation speeder,) and NucS11 (elongation initiator), four nucleosome states enriched with H3K79me2 mark in the gene body. Interestingly, we observed NucS10 with both H3K79me2 and H3K36me3 marks showed the highest enrichment in exons for all three cell types (Fig. 5A). We then defined a NucS-SE affinity, a ratio of SEs associated with a NucS vs randomized SEs associated with that NucS, to semi-quantitatively determine the association between nucleosome states and SE events. We found that NucS10 again showed a higher SE affinity among all three cell types (Fig. 5B). To further determine the splicing potentiality of SEs, we also developed an empirical equation to quantify the splicing potentiality for each of four nucleosome states, where we assessed the splicing potentiality from three following aspects: (1) Fréchet distance between the nucleosome distribution of reliable SE (rSE) and unreliable SE (urSE) (Additional file 1: Fig. S17); (2) the difference of nucleosome positioning between nucleosomes in rSE and urSE (Additional file 1: Fig. S18); and (3) the normalized counts coefficient of each H3K79me2 related NucS (see the “Methods” section and Eq. 9). Remarkably, we found the potentiality score of NucS10 is the highest among all four H3K79me2 related NucSs (Fig. 5C). Together, our results suggest nucleosomes modified with H3K36me3 and H3K79me2 histone tails might play an important role in influencing the skipping exon processing due to its lowest phasing and a higher degree of positioning.

## Discussion

Despite several existing computational methods for determining epigenetic states, none of them is able to quantitatively examine the relationship of nucleosome organization, histone marks, and genomic regions at a finer nucleosome resolution level. To the best of our knowledge, our NucHMM is the first computational algorithm and tool to identify functional nucleosome states associated with cell type-specific combinatorial histone marks and nucleosome organization. We rigorously trained and tested it on all publicly available MNase-seq and ChIP-seq data of various histone marks in MCF7, H1 and IMR90 cells. We were able to identify 11 cell type-specific functional nucleosome states, each encoded with specific biological meanings (Table 1). Importantly, NucHMM is applicable to train MNase-seq and ChIP-seq of various histone marks in many different cell types.

To test the reliability of NucHMM results, we first compared “Training” module of NucHMM with ChromHMM and Segway to evaluate its performance. We found that both NucHMM “Training” module and ChromHMM/Segway produced similar results in terms of HMM states with distinct combinatorial histone marks (Additional file 1: Figs. S5-6). We then used a simulated nucleosome array coverage signal to verify the fidelity of our methods for calculating the phasing score and spacing value (Additional file 1: Fig. S13). Remarkably, the spacing value calculated directly from the simulation sine function is consistent with the one calculated from NucHMM. The phasing score from the simulated signal is also in line with our knowledge. Finally, we constructed the equation for measuring nucleosome positioning with the validation by reference positioning score (Fig. 4) and Nucleosome Dynamics [65] (Additional file 1: Suppl. Notes and Figs. S19-20).

There are several notable strengths of NucHMM. Firstly, we built directional nucleosome-based observations in the “Initialization” module and used it for the univariate HMM “Training” module. The nucleosome-level observations allow us to annotate the combinatorial histone modifications on the nucleosomes (Additional file 1: Suppl. Notes and Figs. S6A, S7B-D, S8A and S8C) and also to capture the 5′ TSS more accurately (Additional file 1: Fig. S8B). While univariate HMM enumerates each possible combination of histone marks as the possible output of HMM, it more straightforwardly determines whether a particular histone mark occurs in a state compared to a multivariate HMM, which also enhances NucHMM ability to precisely annotate HMM states on a nucleosome. Furthermore, the directionality information provides a more realistic model of the underlying epigenetic patterns and their transitions. Secondly, we employed the “Functioning” module to convert HMM states to functional nucleosome states (NucSs), which are associated with not only combinatorial histone modifications, but also with nucleosome organization features, including nucleosome phasing, spacing and positioning (Additional file 1: Fig. S21). This extra layer of nucleosome organization information expands the features space of genomic states from one dimensional (traditional chromatin states) into two dimensional (functional nucleosome states), which, for the first time, offers an opportunity to genome-wide study the interplay of epigenetic marks-nucleosome organization. But there are few limitations of NucHMM: (1) the initial number of HMM states needs to be estimated at the beginning of NucHMM training, (2) the increased number of states and number of nucleosomes requires more computational and memory resources, (3) the initial assignment of a histone mark within a nucleosome bin may not be very accurate since the overlapping criteria between a nucleosome bin and a histone mark peak is a little bit subjective, and (4) the cutoff threshold of the emission probability in the mark-state matrix is arbitrary for determining whether the histone marks should be included into a state. To mitigate these limitations, future improvements may be focused on implementing a parallel computing framework, optimizing the assignment of histone marks and using a statistical method to devise the initial number of HMM states and to define a cutoff threshold of the emission probability.

Importantly, we were able to associate gene body functional nucleosome states with publicly available RNA-seq to quantitatively measure the splicing potentiality. Our quantitative comparison of the influence of four gene body nucleosome states on SE events revealed that NucS10 has the highest SE potentiality (Fig. 5C). This might due to its higher distribution at the middle gene body (Fig. 2F), its lowest nucleosome phasing (Fig. 3C), and its higher degree of positioning (Fig. 4C), as well as its most enrichment at internal exons (Fig. 5A). Most of the previous studies showed either H3K79me2 or H3K36me3 has a role in regulating alternative splicing [28, 29, 66]. However, our analyses clearly showed that the nucleosomes with both H3K36me3 and H3K79me2 marks might have the most effective influence in co-regulating the skipping exon processing. Our finding may offer new opportunities to interrogate the mechanisms of the functional crosstalk between H3K36me3 and H3K79me2 marked nucleosomes and the skipping exon processing.

## Conclusion

In summary, we developed a novel computational method, NucHMM, for identifying cell type-specific nucleosome states. With NucHMM, we identified 11 distinct functional nucleosome states for MCF7, H1, and IMR90 cell types. We further demonstrated that these functional nucleosome states can be used to quantitatively determine the splicing potentiality of SEs. Our work advances our understanding of chromatin function at the nucleosome level and further offers mechanistic insight into the interplay between nucleosome organization and splicing process.

## Methods

### NucHMM initialization

To remove background noises and decrease the false positive rate of called positioned nucleosomes positioning and peaks of histone marks, we performed quality control (QC) for both MNase-seq and ChIP-seq data by using trim-galore [67]. We used bowtie or bowtie2 to uniquely map the reads to human HG19 reference genome. For MNase-seq data, we used Deeptools [68] to keep fragments within the range of 130–180 bp because of the length of the wrapped DNA of nucleosome plus the linker histone is within this range. We applied iNPS, which smoothed the MNase-seq wave profile with Laplacian of Gaussian convolution, to detect the borders of the nucleosome peaks, and then use a Poisson approximation filtering process to locate the final nucleosomes. We used MACS2 to identify narrow peaks for ChIP-seq of H3K4me1, H3K4me3, and H3K27ac but used EPIC2 to identify broad peaks for ChIP-seq of H3K9me3, H3K27me3, H3K36me3, and H3K9me3 with parameters -bin 100, -fdr 0.05, and -g 2 (or -g 5).

The entire genome was then binned based on detected nucleosomes. An alphabet of 128 (2^7^) observation notations was built by enumerating each possible combination of marks (Additional file 1: Table S5) including no marks. For example, observation 9 (0b0001001) corresponds to the presence of H3K4me3 (1 = 0b0000001) and H3K79me2 (8 = 0b0001000) and the absence of all other marks. We then assigned the converted notations to the bins based on the degree of overlapping between the histone mark’s peak and the nucleosome position. We limited the trained genomic region ranging from − 100Kb upstream to 5TSS (Upstream-TSS), gene body (Gene-body), and + 10Kb downstream of transcription terminal site (TTS) (Downstream-TTS) (Additional file 1: Suppl. Notes) and compiled a set of 19,189 protein-coding genes with the unique 5′TSS from UCSC RefSeq Genes.

### NucHMM training

NucHMM training included two rounds of HMM learning process. In the first round, we empirically chose initial states ranging from 15 to 25 and ran five first-order HMMs for each of them. Each HMM was trained for 300 iterations to ensure the convergence using the Baum-Welch algorithm [69]. We then selected the HMM with the lowest Bayesian Information Criterion (BIC) score. Before the second round training, we removed those states with less than 0.5% of the total nucleosomes in the model from the transition probability and emission probability matrices. To simplify the HMM and maximize its states’ the descriptive power, we used the modified transition probability and emission probability matrices for the second HMM learning process. The resulting HMM was trained with the Baum-Welch algorithm (Additional file 1: Suppl. Notes) for another 200 iterations to achieve the final HMM. The log-likelihood of HMM after each iteration was calculated to ensure to reach the local minimum. We found that 200 iterations were sufficient for this second round HMM to approach the convergence. The Viterbi algorithm was applied to decode HMM states on each nucleosome (Additional file 1: Suppl. Notes). The probabilities of an individual histone mark were calculated by marginalization among all output combinations of marks probabilities. The individual emission probability follows

Eq. 1 $$ {\mathit{\Pr}}_{id}={\sum}_{x=1}^{2^n}\left\{{P}_{(x)}\ \left(x\& id>0\right)\right|\ 0\ \left(x\& id=0\right)\Big\}, $$

where *n* is the number of histone marks, & is bitwise AND operator, and *x* is the output number.

### Nucleosome phasing and spacing

We first processed the Gaussian smoothed nucleosome signals from iNPS to nucleosome state-specific nucleosome array signals. We then averaged nucleosome array signals by the sum of all nucleosome array signals within the nucleosome state divided by the number of the nucleosome arrays. As the resolution of the Gaussian smoothed signal is 10 bp/point in the iNPS result, the initial sample rate of the nucleosome state array signal is 100 (10 bp/point). In order to keep the fidelity and more precisely convert the signals from the genome domain to the frequency domain, we first interpolated the signals and increased the sample rate to 1000 (1 bp/point), and then implemented Welch’s method [70] to make the conversion based on the periodogram spectrum estimates, which was used to calculate the nucleosome phasing score.

For a detailed implementation, we firstly used the Hanning window function *w*(*n*) to divide the nucleosome state array signal *x* into K available frames with M points in each frame. Each frame is represented by

Eq. 2 $$ {x}_m(n)\triangleq w(n)x\left(n+ mR\right),n=1,2,\cdots, M-1,m=1,2,\cdots, K-1 $$

where R is the window hop size.

Then, the periodogram of the *m*^*th*^ frame is given by

Eq. 3 $$ {P}_{x_{m,M}}\left({w}_k\right)=\frac{1}{M}\left|{FFT}_{N,k}{\left({x}_m\right)}^2\right|\triangleq \frac{1}{M}{\left|{\sum}_{n=0}^{N-1}{x}_m(n){e}^{-j2\pi nk/N}\right|}^2 $$

We then averaged the periodograms across the genome. The Welch estimate of power spectral density is given by

Eq. 4 $$ {\hat{S}}_x^W\left({w}_k\right)\triangleq \frac{1}{K}{\sum}_{m=0}^{K-1}{P}_{x_m,M}\left({w}_k\right) $$

The simplified conversion equation between the genome domain and the frequency domain is given by:

Eq. 5 $$ freqc=\frac{fs}{\mathrm{genome}\ \mathrm{length}} $$

where *fs* is the sample rate of the signal.

We finally focused on the power spectrum density within frequency 4–10 Hz, which corresponds to the genome domain range 100–250 bp. We used the highest spectral density value of each nucleosome state in the window and multiplied 1000 as the nucleosome phasing score.

The calculation of the nucleosome spacing value utilizes the distribution of a nucleosome state-specific nucleosome array. We computed all local maxima of the array distribution by the following two rules: (1) for sharp peaks, the local maximum is defined as any sample point whose two direct neighbors have a smaller amplitude, and (2) for flat peaks, the middle point index is considered as the local maximum. We then calculated the average distances between the maxima of peaks as the nucleosome spacing value. To determine the spacing range for a nucleosome within a NucS-specific nucleosome array, we used Eq. 6:

Eq. 6 $$ \left[{\mathrm{Spacing}}_{\mathrm{NucS}}-\mathrm{Interval}\times \left(5+\mathrm{Rank}\ast {\mathrm{Coef}}_{\mathrm{range}}\right),{\mathrm{Spacing}}_{\mathrm{NucS}}+\mathrm{Interval}\times \left(5+\mathrm{Rank}\ast {\mathrm{Coef}}_{\mathrm{range}}\right)\right] $$

where Spacing_NucS_ is the average nucleosome spacing of a NucS; Interval is the order of the nucleosome minus one, e.g., for the second nucleosome in the array, its Interval is one; Rank refers to the rank for each of 11 NucSs based on their phasing scores; Coef_range_ is a user-defined parameter that used to adjust the range with 1 bp as the default.

### Nucleosome positioning

We used two inter-correlated approaches, the “raw reads” reference approach and the “iNPS-derived” approach, to determine the nucleosome positioning (NP) score. We defined the well-positioned nucleosomes would have higher positioning score than fuzzy nucleosomes in both methods. Both approaches were applied with the idea that nucleosome positioning is the geometric-mean of the nucleosome fuzziness and nucleosome occupancy. In the “raw-reads reference” approach, we measured three features: (1) the standard deviation of raw reads, (2) the enrichment of raw reads, and (3) the full width at half maximum of reads peak. The equation of this approach can be described as:

Eq 7 $$ {rNP}_t=\frac{\operatorname{norm}\left({\mathrm{enrich}}_t\right)}{\operatorname{norm}\left({\mathrm{fwhm}}_t\right)+\operatorname{norm}\left({\mathrm{std}}_t\right)} $$

where *t* ∈ {1, 2, ⋯, T} = nucleosome population set, and norm represents the interquartile range normalization.

For example, the numerator should be relatively small for a fuzzy nucleosome while the denominator should be large and make the nucleosome positioning score small. In the “iNPS-derived” approach, we first empirically created nine equations based on iNPS results to calculate the nucleosome positioning. We then used the Pearson correlation method to determine which equation has the highest correlation with the “raw-reads reference” approach. The final determined equation is given by:

Eq. 8 $$ {NP}_t=\frac{\mathrm{height}+{\log}_2\left({\mathrm{pval}}_{\mathrm{peak}}\times {\mathrm{pval}}_{\mathrm{valley}}+1\right)+\mathrm{area}}{3\times \mathrm{width}} $$

where height, width, area, pval_peak_ , and pval_valley_are all from iNPS. Generally, the numerator in the Eq. 8 reflected the occupancy measurements and denominator reflected the fuzziness measurement. Besides, we noticed that the pval_valley_ is abnormally high at the end of the nucleosome array regardless of the shape of the real nucleosome. Thus, we manually replaced all pval_valley_ of the last nucleosome in the array with the median value of the whole pval_valley_ set. All elements in Eq. 8 are also applied interquartile range normalization.

### Splicing potentiality of SE

We assessed SE’s the splicing potentiality associated with each of four NucSs with H3K79me2 mark by measuring the difference of nucleosome organization between the reliable SE group and the unreliable SE group. We first used MISO [71] to identify the potential SE events. The reliable SE events result from applying two rules on the identified potential SE events. Rule 1: *X* + *Y* ≥ *N and Y* ≥ 1, where X, Y are integer counts corresponding to the number of reads in each of these categories, (1,0):X, (0,1):Y. Class (1,0) are reads consistent with the first isoform in the annotation but not the second while class (0,1) are reads consistent with the second but not the first. *N* was the cutoff value derived from X + Y frequency distribution. Rule 2: CI-width > median of CI-width, where CI is the confidence intervals outputted by MISO for each estimate of Ψ. The rest of the potential SE events are then defined as unreliable SE group. We then extracted nucleosome distribution from iNPS results based on the coordinates of the rSE and urSE groups. The difference of nucleosome organization between rSE and urSE groups was then measured by Fréchet distance [72] and nucleosome positioning population (Additional file 1: Suppl. Notes—the pseudocode for calculating Fréchet distance). The following equation calculates splicing potentiality of SE (SPSE):

Eq. 9 $$ {\mathrm{SPSE}}_{S_o}=\operatorname{norm}\left(\mathrm{frdist}\right)\times \operatorname{norm}\Big(\mathrm{abs}\left({\mathrm{diff}}_{\mathrm{nucpos}}\right)\times {\mathrm{coef}}_{\mathrm{event}-\mathrm{counts}} $$

where norm means the results scaling to range [0, 1], frdist is the acronym of Fréchet distance, abs is the acronym of absolute function, diff_nucpos_ represents the difference of median values of NucS rSE and urSE group, and coef_event − counts_ is the normalized event count coefficient.

More specifically, the Fréchet Distance (norm(frdist)) is used to measure the difference of the averaged NucS array signal (containing both nucleosome spacing and phasing measurements) between rSE and urSE group (Additional file 1: Fig. S17). The norm(abs(diff_nucpos_) measured the different of the nucleosome positioning between rSE and urSE group (Additional file 1: Fig. S18). The larger the Fréchet distance and nucleosome positioning implied a higher SE potentiality of the NucS. The last ${\mathrm{coef}}_{\mathrm{event}-\mathrm{counts}}=\operatorname{norm}\left(\frac{\mathrm{number}\ {\mathrm{of}\ \mathrm{NucS}}_{\mathrm{rSE}}}{\mathrm{number}\ \mathrm{of}\ \mathrm{NucS}}\right)$ is used to measure the ‘abundance’ of the NucS in the rSE group.

## Supplementary Information

**Additional file 1.** NucHMM: a method for quantitative modeling of nucleosome organization identifying functional nucleosome states distinctly associated with splicing potentiality: Suppl. Notes, Figures and Tables.

**Additional file 2.** The lists of cell type-specific NucSs-genes for MCF7.

**Additional file 3.** The lists of cell type-specific NucSs-genes for H1.

**Additional file 4.** The lists of cell type-specific NucSs-genes for IMR90.

**Additional file 5.** Review history.

## Data Availability

The datasets analyzed during the current study are available in the GEO repository: GSM1238700 [73], GSM1194220 [74], and GSE21823 [75] and in the ENCODE repository [61] : listed in Additional file 1: Table S1. Our method is implemented as a C++/python package and is freely available at Under GNU General Public License (GPL-v3.0) (https://github.com/KunFang93/NucHMM) [76] with the version used to generate data in this manuscript deposited in zenodo (https://zenodo.org/record/4581548) [77].
