# Supplementary material for: NucHMM: a method for quantitative modeling of nucleosome organization identifying functional nucleosome states distinctly associated with splicing potentiality
Source: Genome Biol. 2021 Aug 26;22:250. doi: 10.1186/s13059-021-02465-1 (PMC8390234; doi:10.1186/s13059-021-02465-1)
Supplement: Supplementary file 1 — Additional file 1. NucHMM: a method for quantitative modeling of nucleosome organization identifying functional nucleosome states distinctly associated with splicing potentiality: Suppl. Notes, Figures and Tables. [file 13059_2021_2465_MOESM1_ESM.docx]

**NucHMM: a method for quantitative modeling of nucleosome organization identifying functional nucleosome states distinctly associated with splicing potentiality**

Kun Fang, Tianbao Li, Yufei Huang, Victor X. Jin

**Supplementary Materials**

**Supplementary Notes**

**Gene-centric genomic regions for training NucHMM**

In this study, we chose a gene-centric genomic region for training NucHMM ranging from -100Kb upstream to 5TSS (Upstream-TSS), gene body (Gene-body) and +10Kb downstream of transcription terminal site (TTS) (Downstream-TTS):


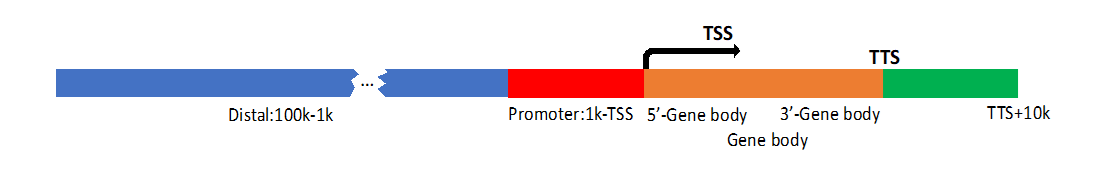


The rationale for choosing such a training region is described in the following. 1) Most of the regulatory elements are harbored within this gene-centric genomic region. Thus, we wouldn’t lose much biological meaningful information from the HMM training. 2) The computing resource is limited so it could save a lot of running time to perform the HMM training. For example, there are over 11 million nucleosomes at a genome-wide in just one cell type, i.e. 11 million nucleosome bins, but only 7 million bins within the chosen region. 3) The inversion of the observation sequence on the negative strand genes is possible. In the fixed bin size-based univariate HMM algorithm, it is impossible to flip over the observation sequence on the negative strand genes as the fixed bins are continuous around the gene boundary. Therefore, the histone marks in the gene body would be trained in HMM before those histone marks on the promoter region, resulting in a systemic error to the model. However, by using the gene-centric genomic region, the bins are ‘discrete’ around the gene boundary. Thus, it allows the inversion of the observation sequence on the negative strand genes.

**Baum-Welch algorithm and Viterbi algorithm in NucHMM**

For a convenience of reference, we define the following model notation for our NucHMM: T = a total number of nucleosomes, $\{1,2,\cdots,t\}$; N = a number of states; M = a number of possible combination of histone marks ($2^{num of histone modi}$); Q = {$q_{1},q_{2}\cdots,q_{N}$}, states; V={$v_{1},v_{2},\cdots,v_{m}$} a set of possible histone marks combination; A = {$a_{ij}$}, $a_{ij}$ = $\Pr\left( q_{j} at t+1 \right|q_{i} at t)$, a distribution of state transition probability; B = $\{b_{j}\left( k \right),b_{j}\left( k \right)=\Pr\left( v_{k} at t \right|q_{j} at t)\}$, a combination of histone marks probability distribution in state j; $\pi=\{\pi_{i}\}$, $\pi_{i}=Pr(q_{i} at t=1)$, randomly initialized state distribution; model parameters $\lambda=(A, B, \pi)$. Each HMM is trained for 300 iterations to ensure that they approach the convergence using the Baum-Welch algorithm. Briefly, the Baum-Welch algorithm updates $\lambda$ in each iteration by 1. $\bar{\pi_{i}}= \gamma_{i}\left( i \right), 1\leq i \leq N$; 2. $\bar{a_{ij}}= \sum_{t=1}^{T-1} \xi_{t}(i, j)/\sum_{t=1}^{T-1} \gamma_{t}\left( i \right)$; 3. $\bar{b_{j}}\left( k \right)= \sum_{t=1, O_{t}=k}^{T} \gamma_{t}\left( j \right)/\sum_{t=1}^{T} \gamma_{t}(j)$, where $\xi_{t}(i, j)$ is the probability of a path being in state $q_{i}$ at nucleosome t and making a transition to state $q_{j}$ at t + 1 nucleosome, given the observation nucleosome sequence and the model $\lambda$, and it can be calculated by equation $\xi_{t}\left( i, j \right)=(\alpha_{t}(i)a_{ij}b_{j}(O_{t+1})\beta_{t+1}(j))/Pr(O|\lambda)$ ; $\gamma_{t}\left( i \right)$ is the probability of being in state $q_{i}$ at nucleosome t, given the observation sequence O and the model $\lambda$ and it can be calculated by equation $\gamma_{t}\left( i \right)= \alpha_{t}(i)\beta_{t}(j)/Pr(O|\lambda)$. Besides, we set a minimum probability of 10^−6^ enforced for the transition and emission probabilities to avoid potential numerical underflow. We then choose the HMM with the lowest Bayesian Information Criterion (BIC) score.

Viterbi decoding algorithm in NucHMM is implemented by the following: Denote $v_{t}(j)$ is the probability that the HMM is in $q_{j}$ after observing the first t nucleosomes and go through the most probable state sequence $q_{1},q_{2},\cdots q_{t-1}$, given the model parameters $\lambda$. ${bt}_{t}(j)$ is Viterbi backtrace which is the best path of HMM in state j at the t nucleosome.

1. Initialization:

$v_{1}\left( j \right)= \pi_{j}b_{j}\left( O_{1} \right); 1\leq j\leq N.$ and ${bt}_{t}\left( j \right)=0; 1\leq j\leq N.$

2. Recursion:

$v_{t}\left( j \right)= {max}_{i=1}^{N}v_{t-1}\left( i \right)a_{ij}b_{j}\left( O_{t} \right); 1\leq j\leq N, 1\leq t\leq T$. And

${bt}_{t}\left( j \right)= {argmax}_{i=1}^{N}v_{t-1}\left( i \right)a_{ij}b_{j}\left( O_{t} \right); 1\leq j\leq N, 1\leq t\leq T$.

3. Termination:

The best score $P*= {max}_{i=1}^{N}v_{T}(i)$

The start of backtrace $q_{T}* = {argmax}_{i=1}^{N}v_{T}(i)$

**The rationale behind removing “redundant states”**

We compared the BIC scores and found that by removing the “redundant states” it decreased the BIC score and generated an improved HMM model (**Fig. S3A**). Besides, by visualizing the first HMM’s state-matrix (**Fig. S3B**) and the “redundant states” switches between first HMM and second HMM (**Fig. S3C**), we found that the “redundant states”, S3/9/12/15/16/10/20, are not associated with any histone modifications, and 99.995% “redundant states” are switched into S8/12 (background states) in the second HMM.

**A validation of Training module of NucHMM**

We validated our Training module with ChromHMM. Since our final model contained 13 states, we also trained 13 states HMM with ChromHMM with the default parameters. In terms of comparing HMM states at the nucleosome level, we annotated nucleosomes with HMM states by the segment files from ChromHMM results. The annotation process was performed by using *bedtools intersect* with no -f and -F parameter involved. As expected, within 13 states, NucHMM and ChromHMM shared 10 states (**Fig. S5**). The criteria for identifying shared states are: 1) in the state-mark matrix (**Fig. S5**), the states should have the same combination of histone modifications; 2) the states should have similar genomic location distribution; 3) we visually inspected some loci to further confirm the results. Ten shared HMM states identified by both NucHMM and ChromHMM are: NucHMM S9 -- ChromHMM S2, NucHMM S4 -- ChromHMM S3, NucHMM S5 -- ChromHMM S4, NucHMM S10 -- ChromHMM S5, NucHMM S2 -- ChromHMM S7, NucHMM S3 -- ChromHMM S9, NucHMM S11 -- ChromHMM S10, NucHMM S13 -- ChromHMM S12, NucHMM S1 -- ChromHMM S13, and NucHMM S7 -- ChromHMM S11.

In addition to a general comparison on HMM training, we also performed a nucleosome-level state comparison. We found that within ‘shared’ HMM states, over an average 85% of nucleosomes are also ‘shared’ (**Fig. S6B-D**). For example, for H1, there are a total of 671,723 and 618,740 nucleosomes that were annotated by ChromHMM S9 and NucHMM S3 respectively. Within those nucleosomes, 613,574 nucleosomes are common (labeled by S9(C)_S3(N) in **Fig. S6C**), which account for 91% and 99% of the annotated nucleosomes by ChromHMM S9 and NucHMM S3, respectively. The common nucleosomes ratio of all ‘shared’ states are 88.5% (ChromHMM), 89.3% (NucHMM) for MCF7, 85.8% (ChromHMM), 90.4% (NucHMM) for H1, and 84.5% (ChromHMM) and 89.0% (NucHMM) for IMR90 (**Fig. S6E** and **Tables S6-S8**).

Taken together, we confirmed that the ‘Training’ module of NucHMM are able to accurately capture the combinatorial histone modification states.

**The Comparison between Training module of NucHMM and ChromHMM/Segway**

ChromHMM: When we performed the analysis in ‘A validation of Training module of NucHMM’ section, we found that there were more than 200K nucleosomes that identified two HMM states by ChromHMM in all three cell types (**Fig. S6A**), which could be a potential pitfall if there is a need to perform a downstream nucleosome organization analysis. Besides the 10 shared states, we found that ChromHMM has two unique states: ChromHMM S6 and S8, while NucHMM identified one unique state: NucHMM S6. The possible reason for missing those two states for NucHMM is that NucHMM used a gene-centric training method in which the distal region of -100Kb upstream TSS might not be long enough to capture such two states. However, NucHMM provides the parameters that can be used to adjust the length of the gene-centric region. While the possible reason for missing one state for ChromHMM is that 13 states model might not be the optimal model for ChromHMM, we trained the other two ChromHMM models with 15 and 17 states. We found that 15-states ChromHMM showed similar states with 13-states model, while 17-states model indeed captured the missing states and expanded some new states from its 13-states model (**Fig. S7A**). However, when we visually inspected newly discovered states, for example E1 in 17-states model, it turned out many annotated E1’s are either 1) not associated with any nucleosomes (**Fig. S7B-C**); or 2) even if associated with nucleosomes but over-annotated to the edge of the histone mark which might belong to another state (**Fig.S7C-D**).

Segway: We also applied Segway to train the data. As we didn’t find Segway’s command that allows training all three cell types together, we trained the data in the individual cell type respectively. We found there are several differences between NucHMM and Segway. Firstly, as Segway runs with 1bp resolution and has to run cell type individually, NucHMM would train the model with a little more efficiency. The training time of NucHMM for the current dataset was ~13h for a single model of all cell types, while Segway needed ~16h*3 with --minibatch-fraction=0.01 and ~50h*3 with –minibatch-fraction=0.1 on our server, and we assumed that the training time for the entire genome would be much longer. Secondly, similar to the comparison between NucHMM and ChromHMM, we found 1223,73, 132,244, 156,709 nucleosomes (**Fig. S8A**) identified with two states by Segway in MCF7, H1 and IMR90 respectively. The screenshots show (**Fig. S8B**) these bi-annotated cases. Further, NucHMM has a little bit larger states change per kb 0.23, 0.22, 0.32 than Segway 0.14, 0.15, 0.20 in MCF7, H1 and IMR90. Finally, in the 13-states DBN model, we found that the states identified by Segway are tended to have more high-probability marks than NucHMM, which made it a little bit more difficult for users to tell the difference between the states.

However, we recognized that the model from Segway might not be the optimal one that Segway might be able to learn since many parameters in Segway could be fine-tuned. For example, 1) we only used one initial seed to train DBN (not set --num-instances), which only represents a local-optimal network for the data; 2) we only used the minibatch parameter (0.1) to train the network to speed up the training process, which might not reflect the optimal result that trained by the entire genome; 3) for the purpose of the comparison, we used the graph with 13 nodes which might not be the optimal graph network nodes; and 4) we only applied the default parameters to train the network although Segway has many other parameters for fine-tuning.

In summary, Training module of NucHMM showed improved combinatorial histone modifications annotation on the nucleosomes than ChromHMM and Segway, in which it is more suitable as the input for performing the nucleosome organization analysis in Functioning module.

**Verifications of calculation methods for the phasing score and spacing value**

We verified our calculation method by simulating a 1000 bp nucleosome array coverage. The signal of the simulation data was acquired from the following sine function:

$$y=0.8\sin\left( 2\pi*f*\frac{x}{Fs} \right)+2.2$$

, where f = 6 represents the number of nucleosome signal peaks in the regions and Fs = 1000 represent 1000 bp region.

The verification for the spacing calculation: from the sine function, we can calculate the period of the function is:

$$period= \frac{2\pi}{2\pi*\frac{f}{Fs}}= \frac{Fs}{f}=166.6 bp$$

, which is closed with NucHMM spacing observation 167.5 bp.

The verification for the phasing score calculation: It is well-known that a sine function is phased. In the **Fig. S13B**, we showed that the NucHMM calculated phasing score of the simulated signal are the highest and about 3.5 times higher than S9.

**The comparison of the positioning score between NucHMM and Nucleosome Dynamics**

We compared NucHMM with the Nucleosome Dynamics (nucleR is included). We noticed that Nucleosome Dynamics assigned multiple nucleosomes to a wide range signal while the underneath nucleosome calling program of NucHMM, iNPS, annotated this wide region as a single nucleosome. Thus, in the following comparison, we excluded those one-to-multiple mapping nucleosomes.

We first investigated the correlation of score_width and score_height between Nucleosome Dynamics and NucHMM. As there is no score_width and score_height in the iNPS, we thus considered sqrt(1/width*pval-valley) as score_width and sqrt(height*pval-peak) as score_height for comparison purpose. We found that there is relatively high correlations between two programs: r_score_width_ = 0.85,0.82,0.83 ; r_score_height_ = 0.94,0.95,0.95 for MCF7, H1, IMR90, respectively (**Fig. S23A**). However, the positioning scores between the two programs show a relatively poor correlation r_positioning_score_ = 0.60,0.69,0.72 (**Fig. S23B**). The potential reason would be NucHMM defined the positioning score by an idea of the geometric-mean while Nucleosome Dynamics defined the positioning score by the arithmetic-mean. We then calculated the positioning score in the arithmetic-mean way to verify our guess and it indeed showed a more strong correlation r = 0.88,0.87,0.85, for MCF7, H1, IMR90, between the two programs (**Fig. S23C**). In the NucHMM, we would like to use geometric-mean way as its tends to provide a bigger difference (mean_arithmetic_ ≥ mean_geometric_) for the well positioned nucleosome and the poor positioned nucleosome. For example, a well-positioned nucleosome with score_width = 0.9 and score_height = 0.9 would have a positioning score 0.9 for both geometric mean and arithmetic mean, but for a poorly positioned nucleosome with score_width = 0.1 and score_height = 0.9, the geometric mean = 0.3 while the arithmetic mean = 0.5. We also attached some screenshots from MuGVRE [75] and showed the positioning score correlated well between NucHMM and Nucleosome Dynamics for well-positioned nucleosomes but had some differences between poorly-positioned nucleosomes (**Fig. S24**).

**The description of functional nucleosome states in Table 1**

We were able to define 11 functional nucleosome states, NucS1-NucS11 (**Table1)**.

NucS1 is defined as an Elongation accelerator as it is associated with the sole H3K79me2 and mainly distributed at 5’ gene body region.

NucS2 is defined as a Compacting organizer because it has the highest average nucleosomes in an array of 8.49, the 2^nd^ largest average spacing of 195 bp, the modest phasing score of 10.82 and is enriched with H3K9me3 mark.

NucS3 is defined as an Elongation stabilizer for it is related to the sole H3K36me3 and located at 3’ gene body, and notably has a relative larger nucleosome spacing and low phasing score.

NucS4 is defined as an Accessible booster due to its mainly locating in the distal region and having an average 4 nucleosomes in an array, a shorter spacing (3^rd^ shortest spacing) and relatively low phasing score of 10.96.

NucS5 is defined as a Primed intermediator because of its locating in the distal/promoter regions and its enriching with primed or poised H3K27me3/K4me1marks.

NucS6 is defined as an Elongation terminator since it is mainly enriched at the 3’ gene body and associated with H3K4me1/K36me3 marks, as well as has the 2^nd^ shortest spacing and phasing score.

NucS7 is defined as an Elongation processor since it is correlated with H3K4me1/K36me3/K79me2/K9me3/K27me3 marks and presented at the gene body, as well as interestingly has the highest average nucleosome positioning score with a low phasing score.

NucS8 is defined as a Transcriptional stimulator because its well-positioned array is located around the promoter region with active H3Kme3/K4me1/K27ac marks, and has the shortest nucleosome spacing and the lowest nucleosome positioning score.

NucS9 is defined as a Crowding controller because of its higher number of average positioned nucleosomes in an array of 7.37 and its largest average spacing of 196.75 bp, the 2^nd^ highest phasing score as well as its enriching with H3K27me3 mark.

NucS10 is defined as an Elongation speeder for it is enriched with H3K36me3/K79me2 marks and distributed in the gene body, and has the lowest phasing score.

NucS11 is defined as an Elongation initiator because it is associated with H3K4me3/K4me1/K27ac/K79me2 marks and mainly located at 5’ gene body and promoter regions, as well as has the modest spacing, positioning and phasing.

**Cell type-specific NucSs-genes analysis**

To identify cell type-specific genes that have the differential NucS annotation, we performed the following analysis with four steps:

1) We constructed a NucS array signal matrix (${NSM}_{NucS}^{celltype}$) for NucSs in each cell type. Rows in the ${NSM}_{NucS}^{celltype}$ represent genes and columns represent the NucS-associated genomic region. We first mapped the NucS array signal to each of all refseq genes with a binary annotation. Binary annotation means if a base pair is covered by a NucS array, this base pair will convert to 1 from 0. Then, we looped through this process for all genes and all NucSs in each of three cell types to build a total $11\times3 ({num}_{NucS}\times{num}_{celltype}) {NSM}_{NucS}^{celltype}$. We rescaled all matrices to 1000 columns with each column represent 100 bp length for the purpose of the comparison and easy visualization.

2) Cell type-specific (or differential) NucS array signal matrix (${DNSM}_{NucS}^{celltype}$) was calculated by

${{DNSM}_{NucS}^{celltype}= DNSM}_{NucS}^{celltype}- \sum{DNSM}_{NucS}^{other\_celltype}$;

3) The NucS array signal fold change (FC) of each gene was then calculated by $FC= \frac{colsum({DNSM}_{NucS}^{celltype})}{number of columns}$. And log10(FC) value was also calculated and one-way ANOVA analysis was performed across three cell types to calculate the p-value. To test whether the NucS array signal of a particular gene is significantly different across the three cell types, we first multiply the ${DNSM}_{NucS}^{celltype}$ with the location index matrix. Location index matrix is a matrix that store the index information for each row element. For example, for a array [0,0,0,1,1], the location index array is [1,2,3,4,5]. The reason why we multiplied the location index matrix is that not only the number of the NucS signal matters, but also the location of the signal matters. For example, for a pure statistical ANOVA test, these three arrays [0,0,0,0,1], [1,0,0,0,0] and [0,0,1,0,0] are the same, but in a biological aspect, these three arrays should be slightly different as the NucS signal shift two positions. Therefore, by multiplying with the location index array [1,2,3,4,5], the signal array will convert to [0,0,0,0,5], [0,0,0,0,0] and [0,0,3,0,0] and the ANOVA test would recognize them as different arrays.

4) Finally, cell type-specific NucSs-genes was selected by the cutoff log10(FC) >=1 and p-value <= 0.05. Please note that log10(FC) cutoff =1 was empirically selected to make sure the NucS arrays signal in this cell type is at least ten times stronger than other cell types.

**Other eight tested equations for calculating nucleosome positioning**

- Equation 1: ${NP}_{t}=\frac{height+{log}_{2}\left( {pval}_{peak}+{pval}_{valley}+1 \right)+\sqrt{area}}{2\times width}$
- Equation 2: ${NP}_{t}=\frac{height+{pval}_{peak}+{pval}_{valley}}{3\times width}$
- Equation 3: ${NP}_{t}=\frac{height+{pval}_{peak}+{pval}_{valley}+area}{4\times width}$
- Equation 4: ${NP}_{t}=\frac{height+\sqrt{\left( {pval}_{peak}+{pval}_{valley} \right)}+area}{3\times width+\sqrt{width}}$
- Equation 5: ${NP}_{t}=\frac{height+\sqrt{\left( {pval}_{peak}+{pval}_{valley}+area \right)}}{2\times width+\sqrt{width}}$
- Equation 6: ${NP}_{t}=\frac{height\times\sqrt{\left( {pval}_{peak}+{pval}_{valley} \right)}\times area}{{width}^{3}}$
- Equation 7: ${NP}_{t}=\frac{height+{log}_{2}\left( {pval}_{peak}+{pval}_{valley}+1 \right)+area}{3\times width}$
- Equation 9: ${NP}_{t}=\frac{height\times{log}_{2}\left( {pval}_{peak}\times{pval}_{valley}+1 \right)\times area}{{width}^{3}}$

**Pseudocode of Fréchet distance**

Function dF(*P, Q*): real;

input: polygonal curves P = ($u_{1}$, . . . , $u_{p}$) and Q = ($v_{1}$, . . . , $v_{q}$).

return: $\delta_{dF}$ (*P, Q*)

ca : array [1..*p*, 1..*q*] of real;

Function c(*i, j*): real;

if ca(*i, j*) > −1 then return ca(*i, j*)

elseif *i* = 1 and *j* = 1 then ca(*i, j*) := d($u_{1}$, $v_{1}$,)

elseif *i* > 1 and *j* = 1 then ca(*i, j*) := max{ c(*i* − 1, 1), d($u_{i}$,$v_{1}$) }

elseif *i* = 1 and *j* > 1 then ca(*i, j*) := max{ c(1, *j* − 1), d($u_{1}$, $v_{j}$) }

elseif *i* > 1 and *j* > 1 then ca(*i, j*) :=

max{ min(c(*i* − 1, j), c(*i* − 1, *j* − 1), c(*i*, *j* − 1)), d($u_{i}$, $v_{j}$ ) }

else ca(*i, j*) = $\infty$

return ca(i, j);

Start

for *i* = 1 to *p* do for *j* = 1 to *q* do ca(*i, j*) := −1.0;

return c(*p, q*);

End.

**Supplementary Figures**

**Figure S1 Genomic Location Definition**

**Fig. S1.** The log-likelihoods and BIC scores of 13-state HMM derived from 20-state HMM at the 2nd round HMM iteration.

**Fig. S2.** The gap length (nucleosome spacing) distribution of three cell types. Only very small percentage of the pairs of nucleosomes with a spacing larger than 512 (= 2^9^) bp. In NucHMM, by default, if two nucleosomes have a gap larger than 350 bp, they will be assigned into different nucleosome arrays even if they belong to the same HMM state.

**Fig. S3**. A. The BIC scores of 1^st^ HMM training (iteration 0-300) and 2^nd^ HMM training (iteration 300-500). B. The line plot showing seven states are redundant in the current ‘best’ model. This plot included S10, which is not shown in Fig. 2B as we wanted to provide a ‘zoom-in’ view for those low frequency states in Fig. 2B. C. The 1^st^ HMM state mark matrix. D. The “redundant states” switches between 1^st^ HMM training and 2^nd^ HMM training. 99.995% (220,258 out of 220,271) “redundant states” were merged into S8/12 (background states) in the 2nd HMM training, only with 3 nucleosomes from S3 to S9 and 10 nucleosome from S9 to S9. Left nodes: “redundant states” in the 1^st^ HMM training; Right nodes: “background state” in the 2^nd^ HMM training.


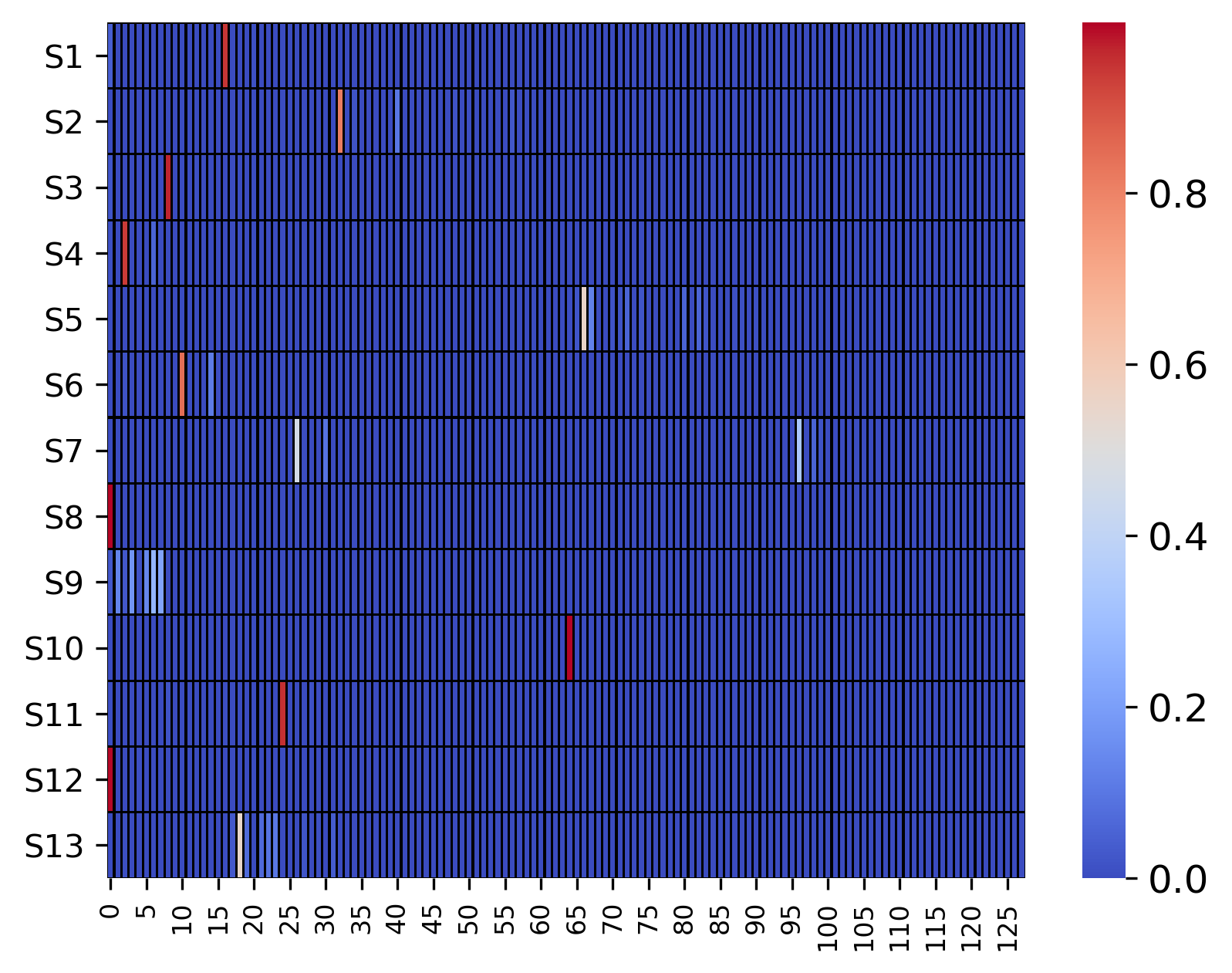


**Fig. S4.** An emission probability matrix for the 13-states HMM model.

**Fig. S5.** Mark-state matrix comparison between ChromHMM and NucHMM. There were ten HMM states that were identified in both ChromHMM (C) and NucHMM (N): S2(C)-S9(N), S3(C)-S4(N), S4(C)-S5(N), S5(C)-S10(N), S7(C)-S2(N), S9(C)-S3(N), S10(C)-S11(N), S11(C)-S7(N), S12(C)-S13(N), S13(C)-S1(N). There were two states that were uniquely identified by ChromHMM: S6(C) and S8(C), and one state that was uniquely identified by NucHMM: S6(N).

**NucHMM**

K4me3

K4me1

K27ac

K36me3

K79me2

K9me3

K27me3

S1

S2

S3

S4

S5

S6

S7

S8

S9

S10

S11

S12

S13

0.8

0.6

0.4

0.2


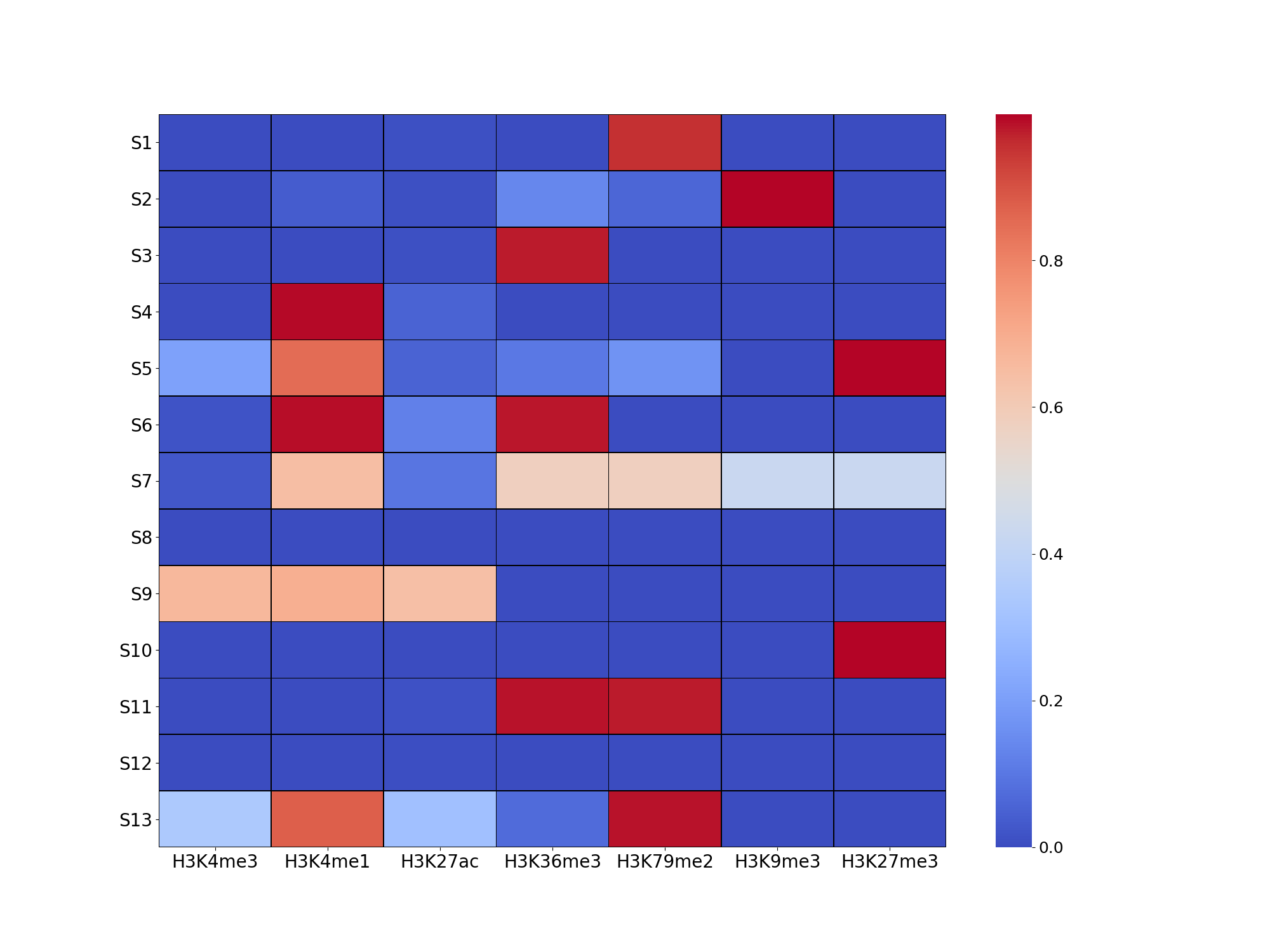


**ChromHMM**


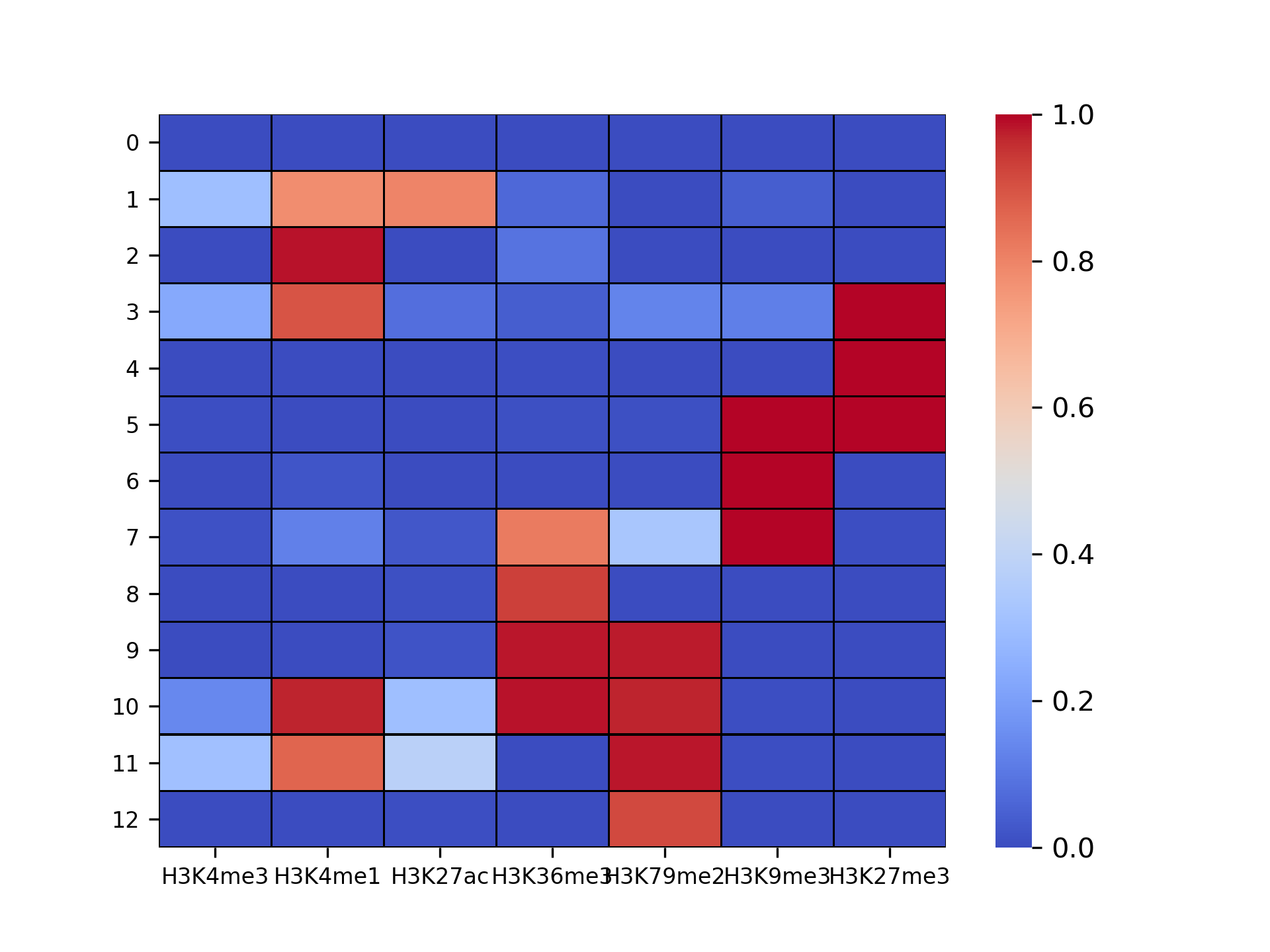


K4me3

K4me1

K27ac

K36me3

K79me2

K9me3

K27me3

S1

S2

S3

S4

S5

S6

S7

S8

S9

S10

S11

S12

S13

**Fig. S6.** A nucleosome-level comparison between ChromHMM and NucHMM. A. There are 210,728, 204,329, and 268,339 nucleosomes crossing the boundaries of the two HMM states identified by ChromHMM for MCF7, H1 and IMR90 respectively, while NucHMM doesn’t have any nucleosomes showing crossover the state boundary. B. The number of common and unique nucleosomes that were annotated by ‘shared’ HMM states in MCF7. C. The number of common and unique nucleosomes that were annotated by ‘shared’ HMM states in H1. D. The number of common and unique nucleosomes that were annotated by ‘shared’ HMM states in IMR90. E. The Sankey plot visualizes the states comparison results.

**Fig. S7.** The comparison with ChromHMM. A. 15 and 17-states ChromHMM, B-C. IGV screen shots show the some E1 states identified in 17-states ChromHMM are E1’s are not associated with any nucleosomes. C-D. Wrongly annotated on the nucleosomes.

**Fig. S8.** A. There are 122,373, 132,244, and 156,709 nucleosomes crossing the boundaries of the two HMM states identified by Segway for MCF7, H1 and IMR90 respectively. B. NucHMM precisely identified 5’TSS with a clear NFR, in comparison to Segway and ChromHMM. C. The IGV screenshots show a scenario that the nucleosomes are bi-annotated by the ChromHMM and Segway.

**Fig. S9.** Distributions of cell type-specific states in the upstream 10kb to TTS region for MCF7, H1 and IMR90 cells respectively.


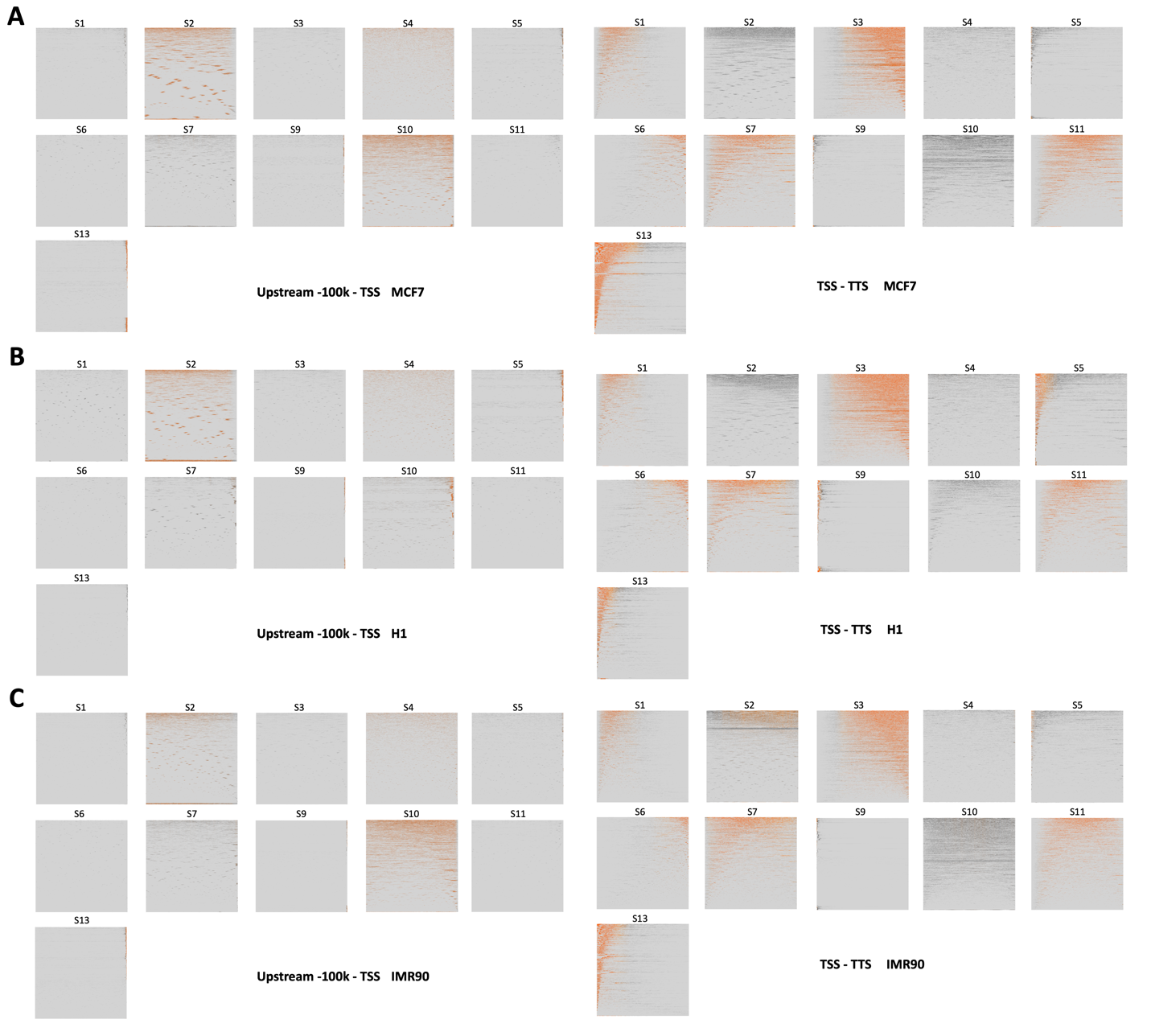


**Fig. S10.** The heatmaps of the states in the upstream 100kb to TSS and TSS to TTS regions for MCF7, H1, IMR90 cells respectively.

**Fig. S11.** Distributions of cell type-specific states in TTS -4kb to TTS +4kb regions for MCF7, H1 and IMR90 cells respectively.

**Fig. S12.** A plot of states' spectral density.


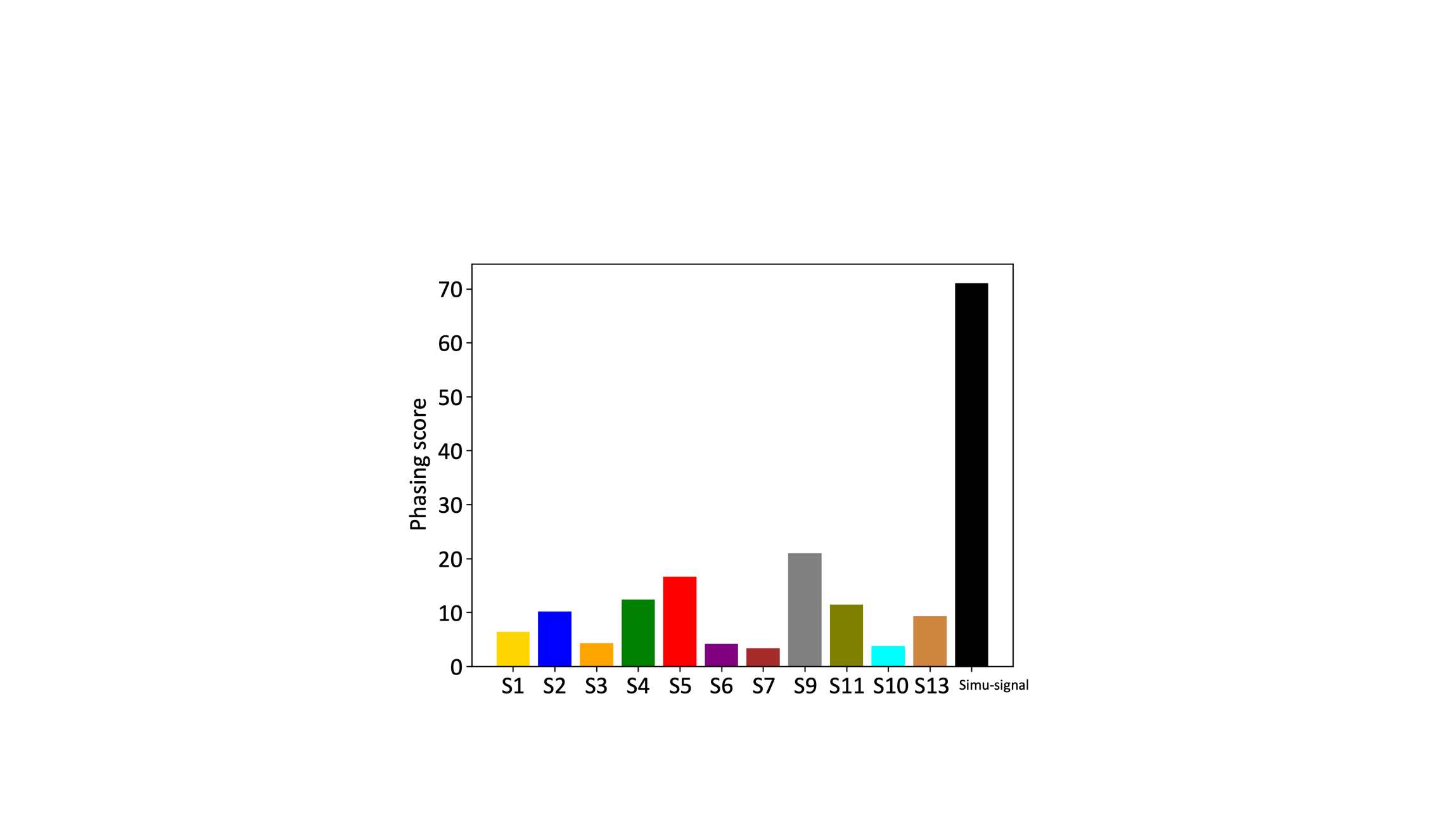

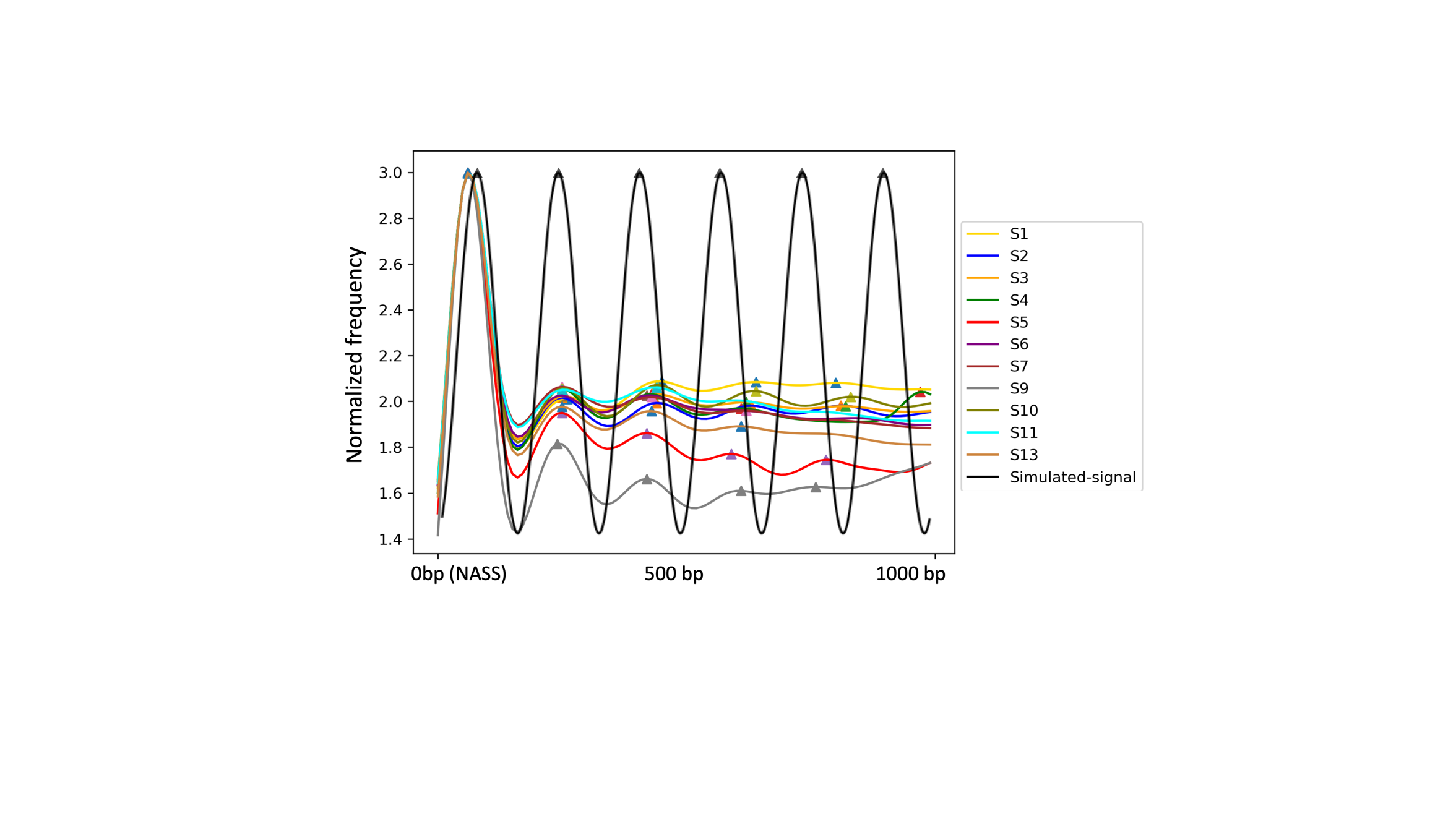


**Fig. S13B.** A bar plot showed the phasing score of each HMM state and simulated signals.

**Fig. S13A.** A Nuc-array-coverage plot of states and simulated phased signals.

**Fig. S14B.** Nucleosome Positioning Scatter plot for IMR90 cells.

**Fig. S14A.** Nucleosome Positioning Scatterplot for H1 cells.


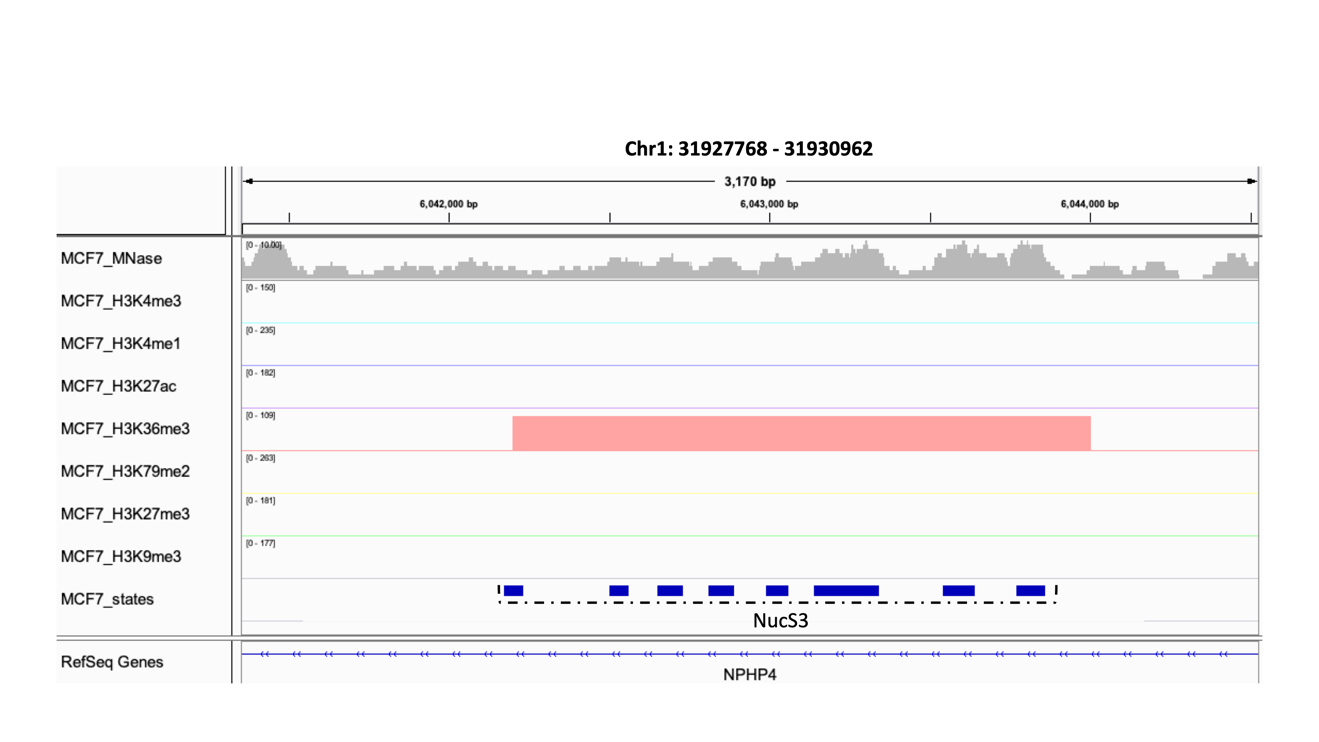

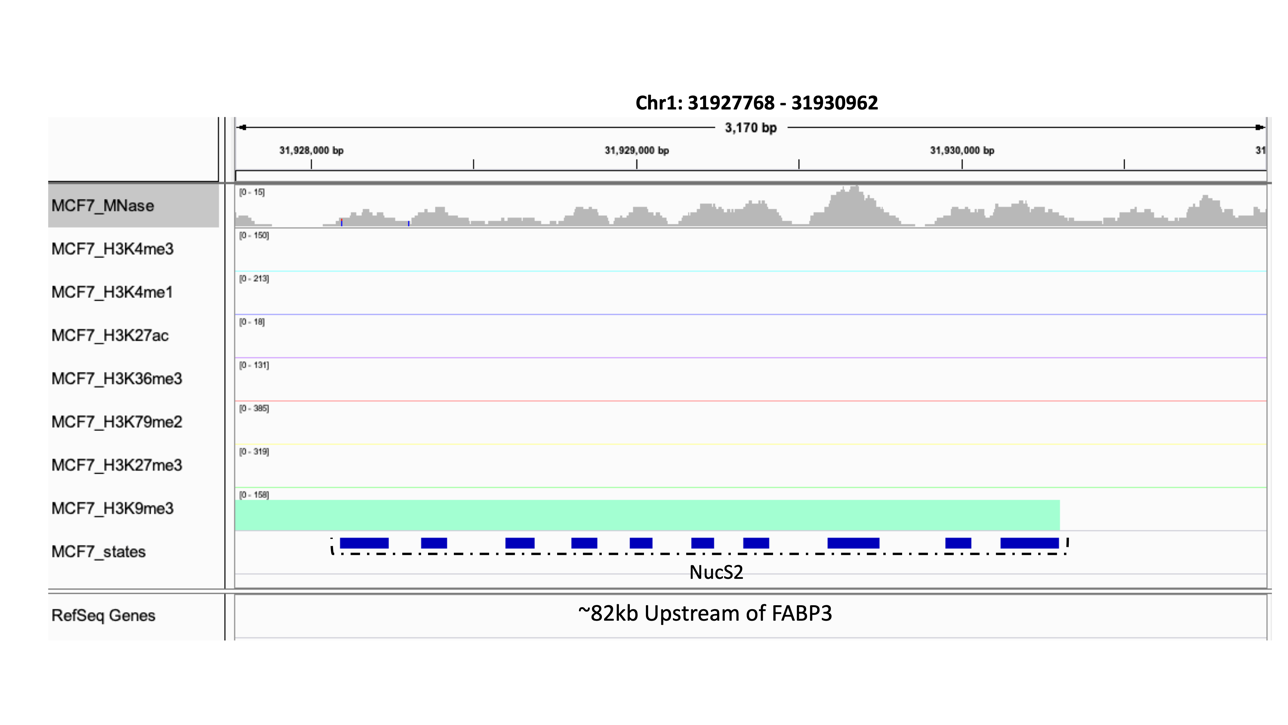

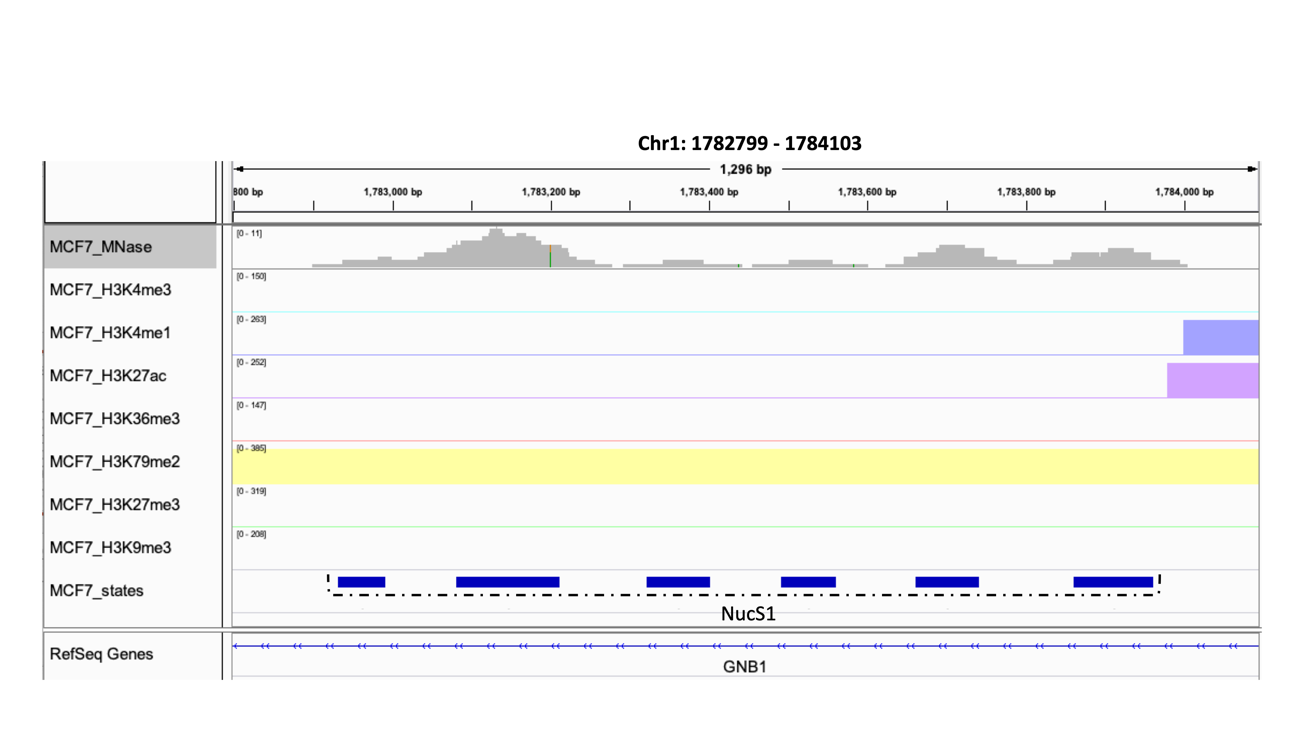


**Fig. S15.** The IGV screenshots of the NucS1/NucS2/NucS3 with histone marks and gene relevance tracks (continue in the next page).


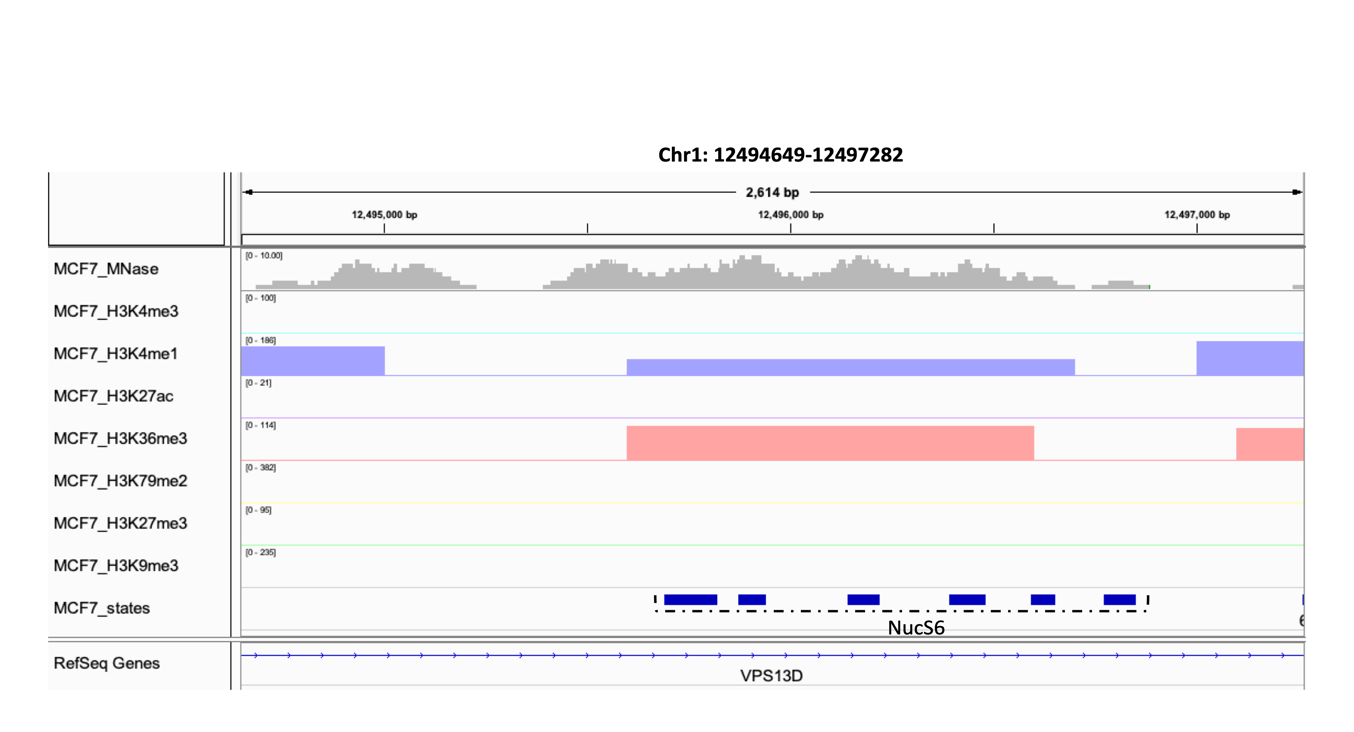

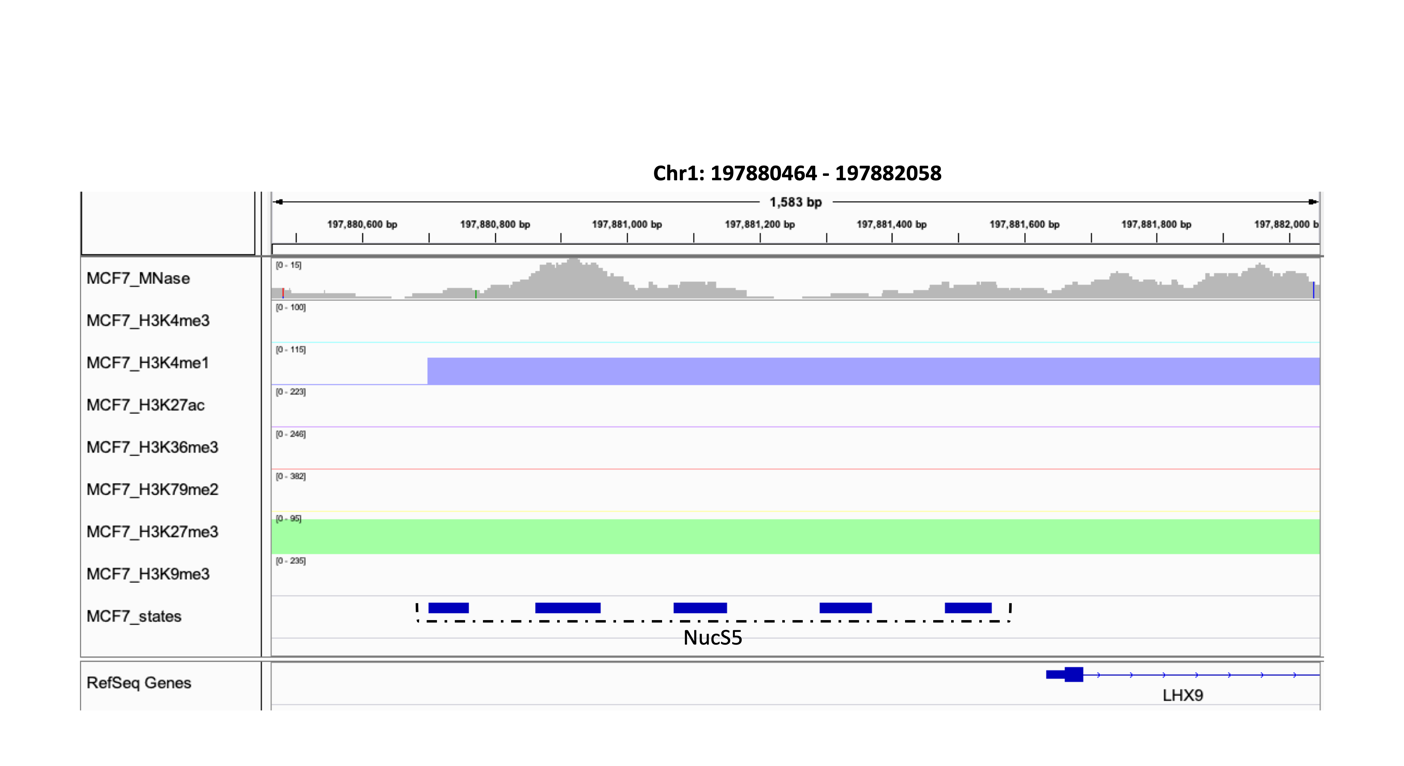

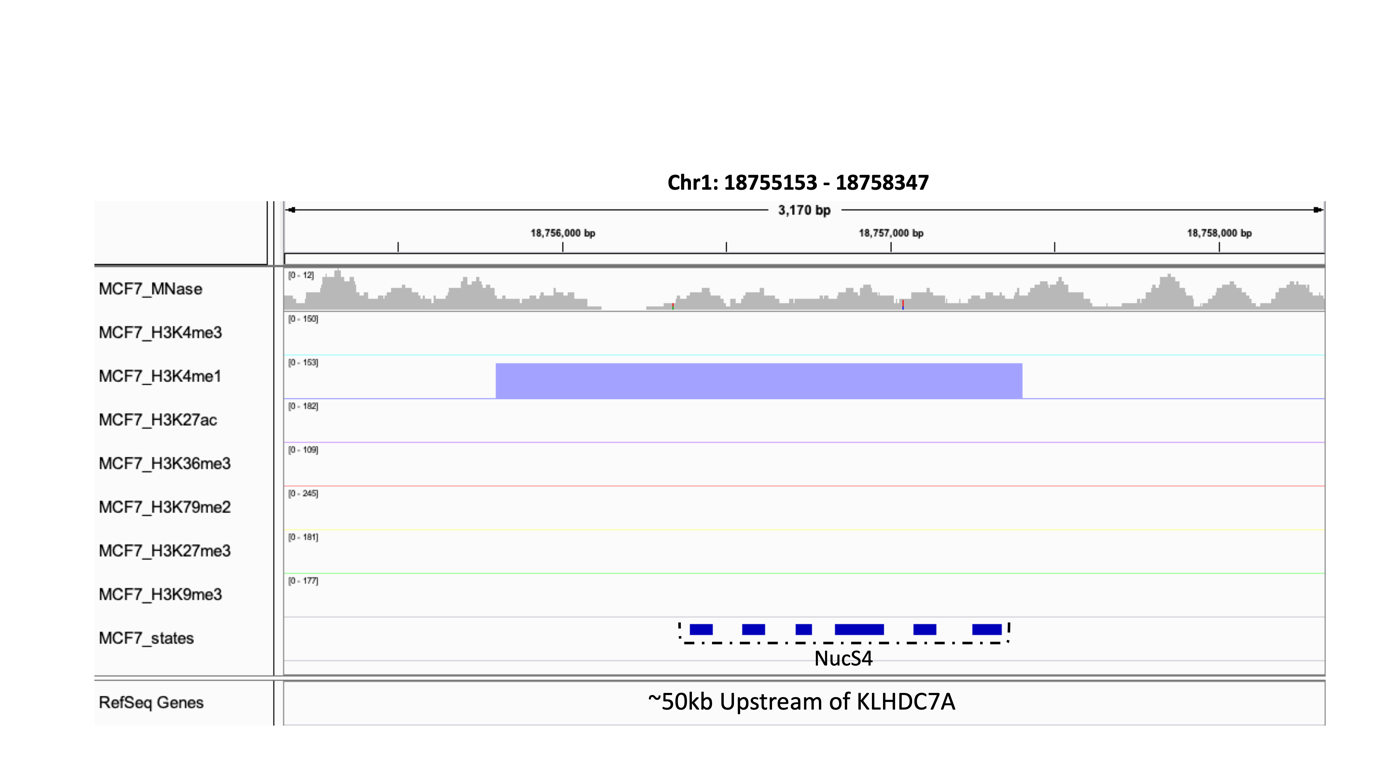


**Fig. S15 (continued).** The IGV screenshots of the NucS4/NucS5/NucS6 with histone marks and gene relevance tracks (continue in the next page).


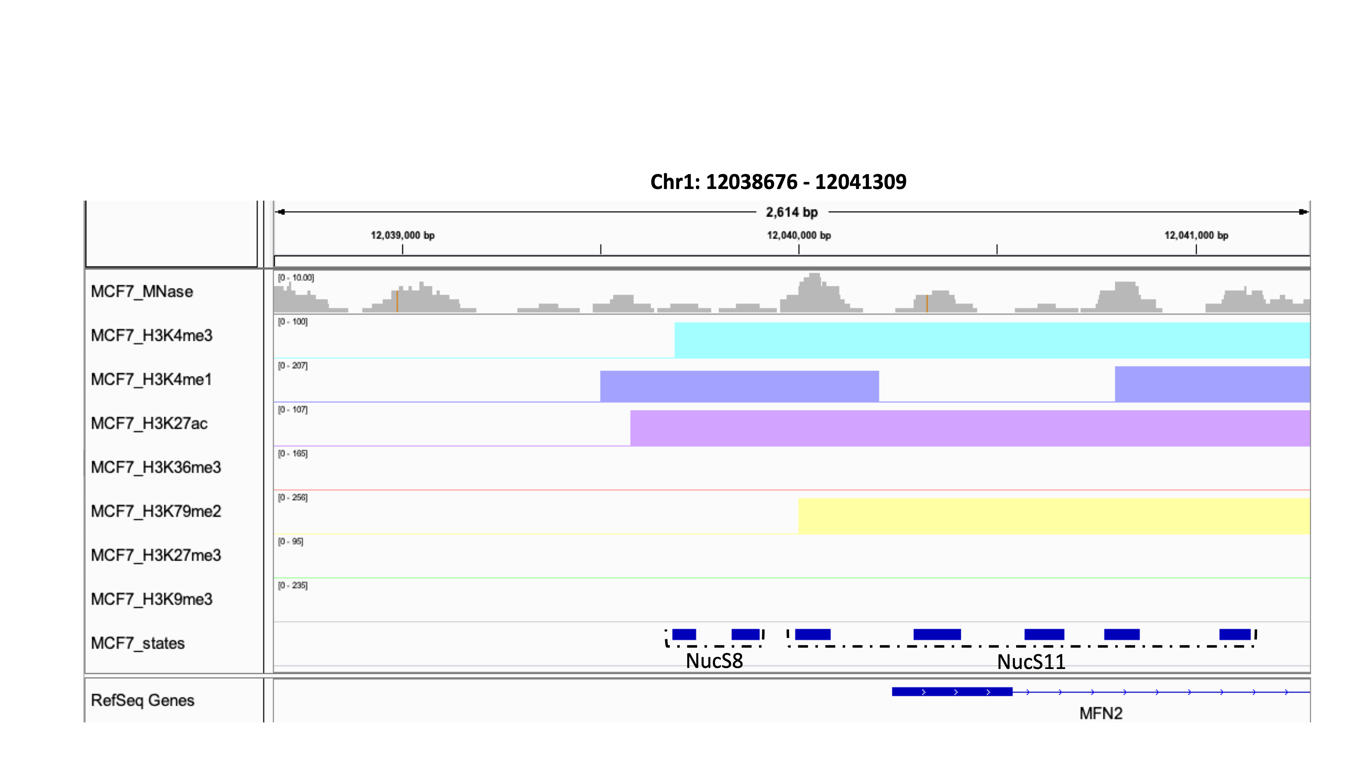

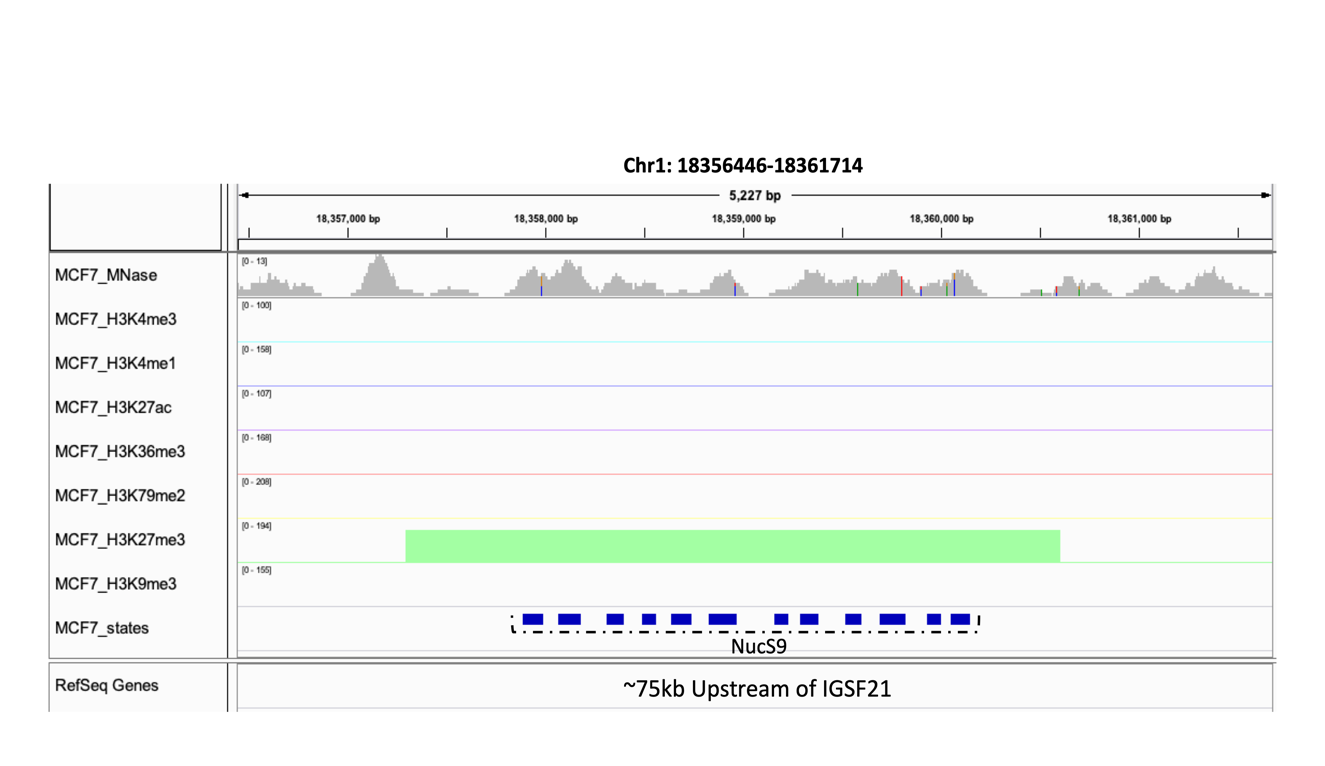

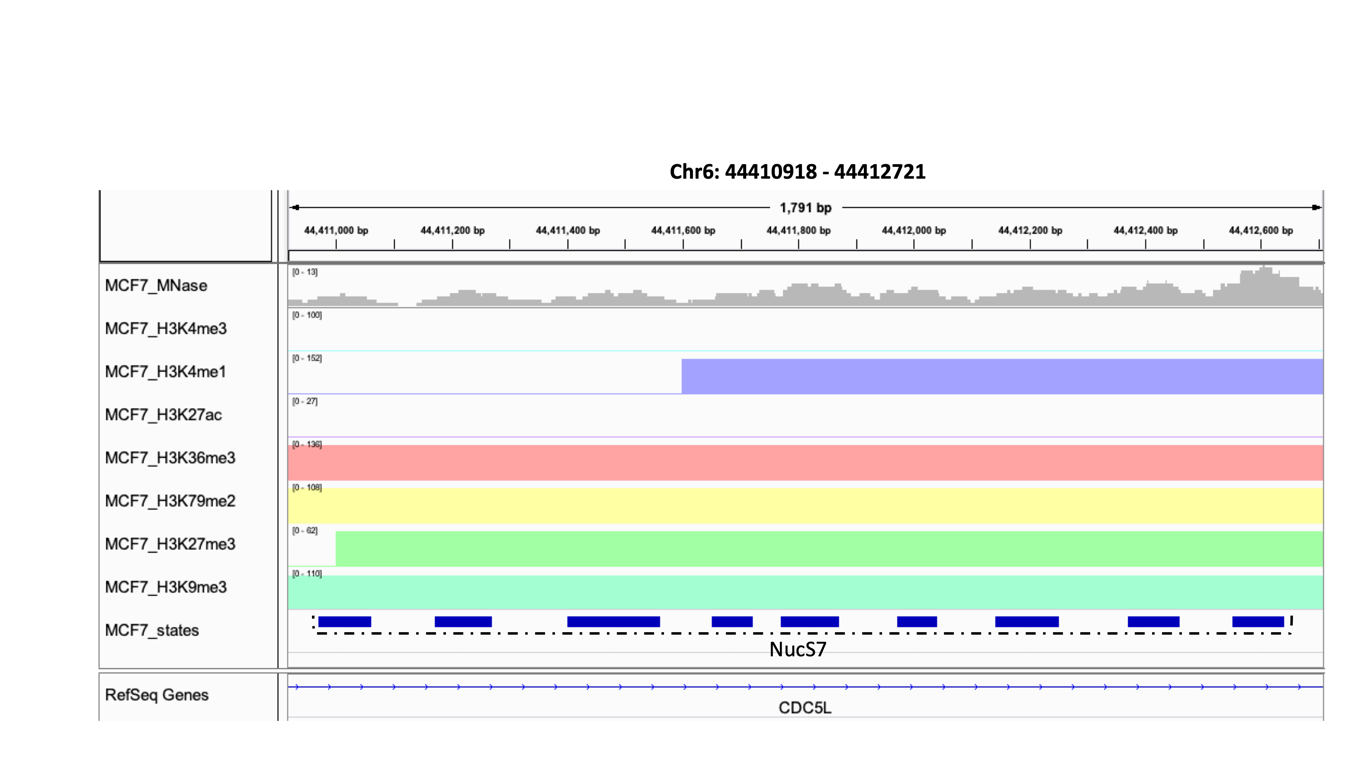


**Fig. S15 (continued).** The IGV screenshots of the NucS7/NucS8/NucS9 with histone marks and gene relevance tracks (continue in the next page).


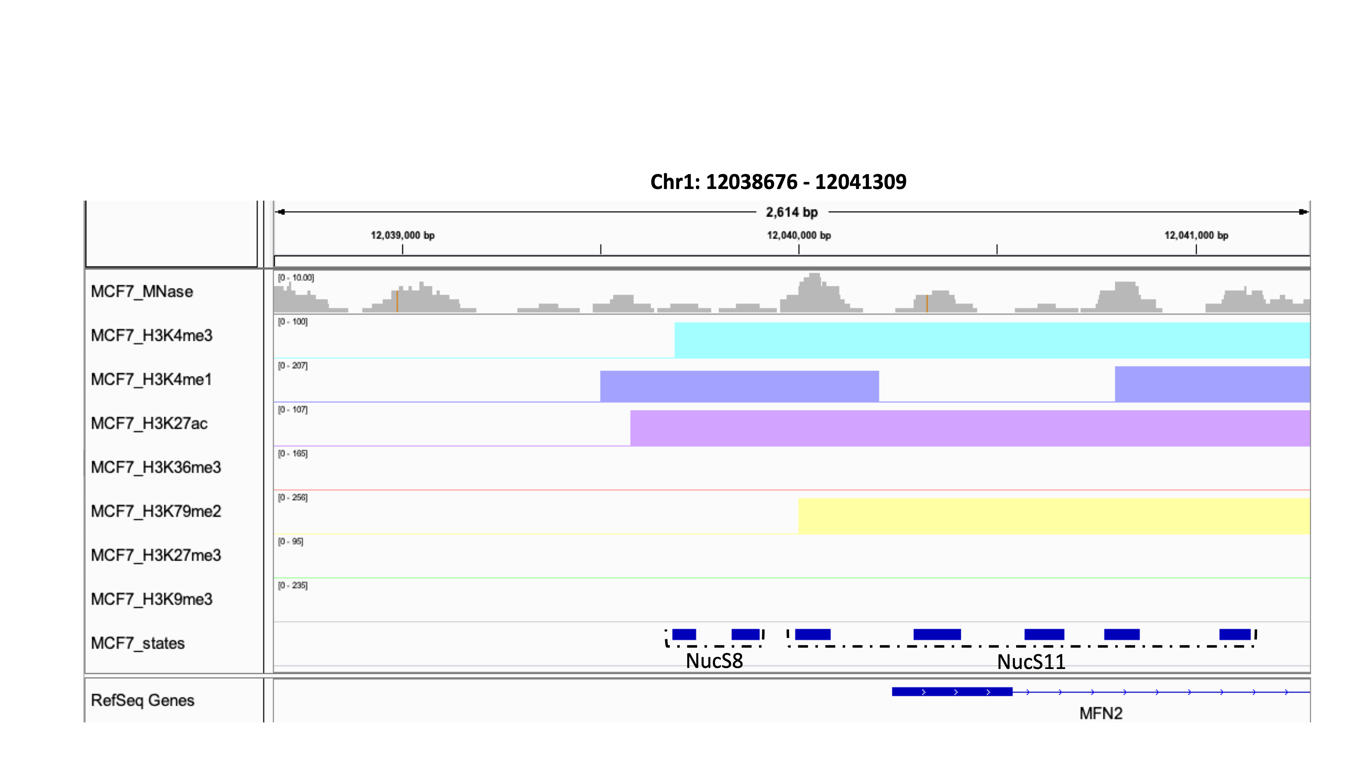


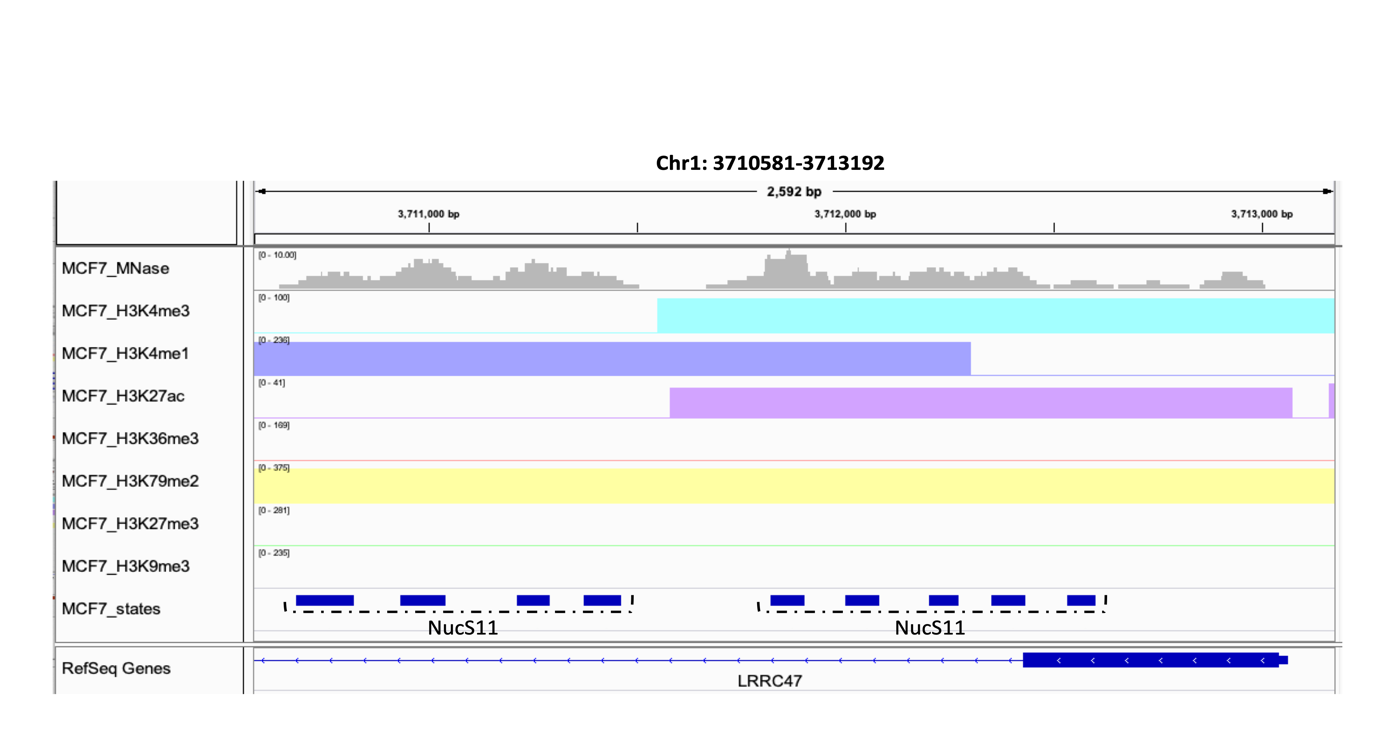

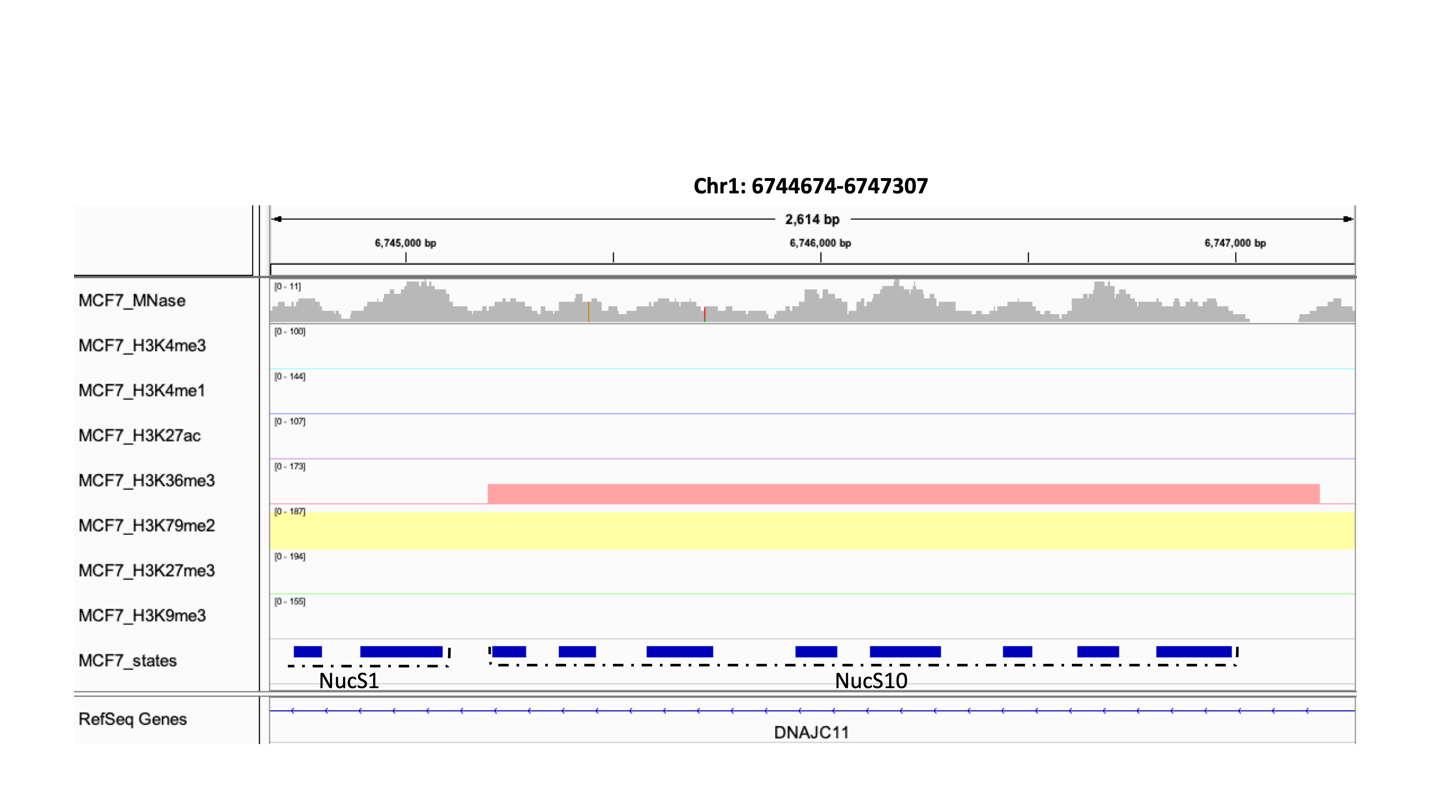


**Fig. S15 (continued).** The IGV screenshots of the NucS10/NucS11 with histone marks and gene relevance tracks.

**Fig. S16**. Cell type-specific NucSs-genes analysis. For each plot with a NucS, the heatmap (left panel) showed the differential NucS array signal in the genomic region associated with that NucS, and the bar plot (right panel) showed a log10(FC) of the NucS array signal for each of the Refseq genes.

**Fig. S16 (continued)**. Cell type-specific NucSs-genes analysis. For each plot with a NucS, the heatmap (left panel) showed the differential NucS array signal in the genomic region associated with that NucS, and the bar plot (right panel) showed a log10(FC) of the NucS array signal for each of the Refseq genes.

**Fig. S17.** Plots of averaged signals at the rSE(urSE) event mid $\pm100 bp$ region. Dash box shows the Fréchet Distance between rSE and urSE in the dash box region. 1/7/11/13 refer to NucS1/NucS7/NucS10/NucS11.

**Fig. S18.** A bar plot of nucleosome positioning for the potential SE associated states. 1/7/11/13 refer to NucS1/NucS7/NucS10/NucS11


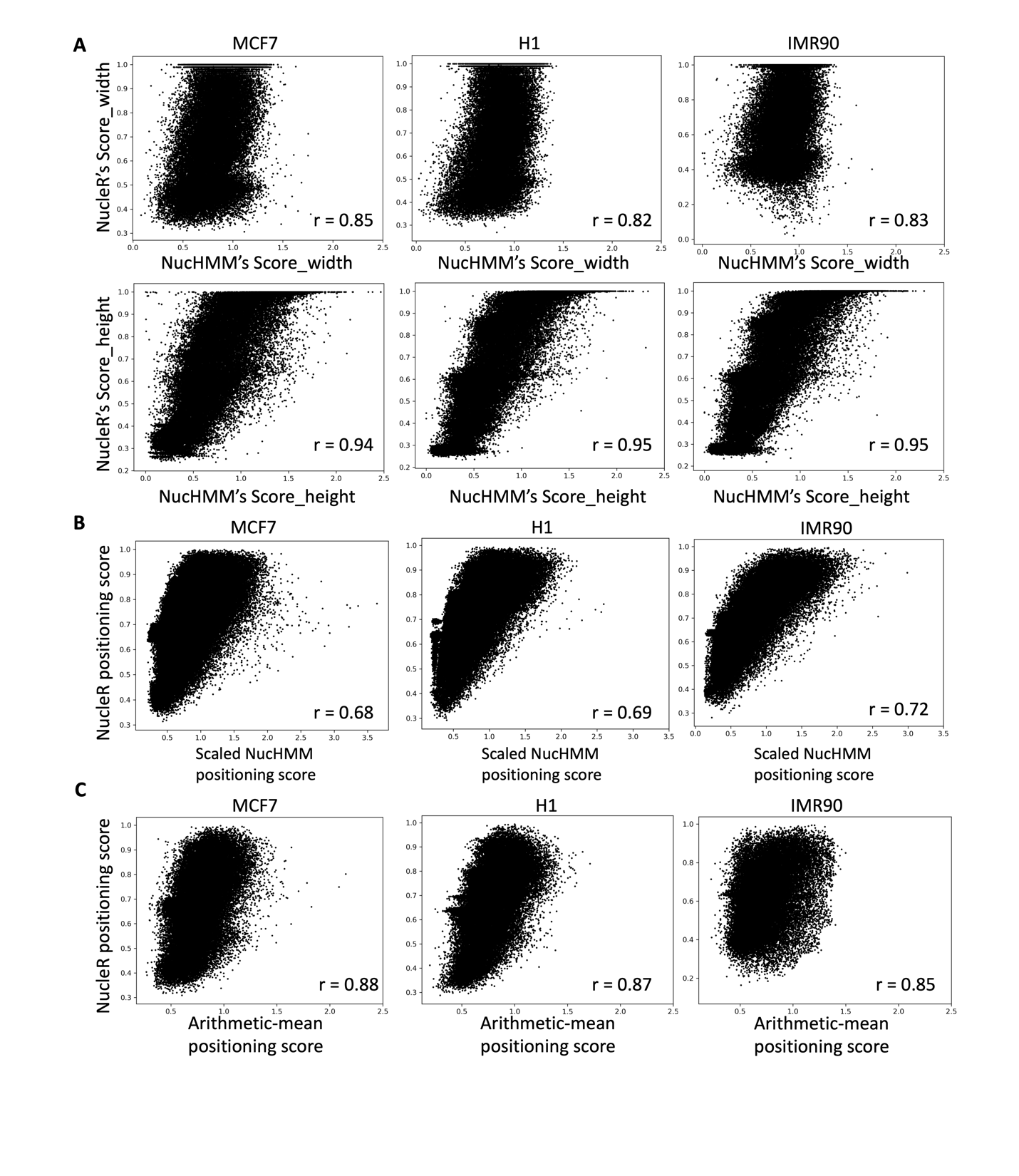


**Fig. S19. The comparison between Nucleosome Dynamics (NucleR) and NucHMM**. A. The scatterplot shows the correlation of score_width (upper panel) and score_height (bottom panel) between two programs. B. The scatterplot shows the correlation of positioning score between two programs. C. The scatterplots shows the correlation of the arithmetic-mean positioning score of NucHMM and Nucleosome Dynamics.

**Fig. S20.** The visualization of nucleosome positioning score of the NucHMM and Nucleosome Dynamics by MuVGRE**.**

**Supplementary Tables**

**Fig. S21.** The IGV screenshots show few cases about how Functioning module works**.**

**Table S1**. Datasets used in the NucHMM training and Splicing potentiality measuring:

|  | MCF7 | H1 | IMR90 |
| --- | --- | --- | --- |
| H3K4me3 | ENCSR985MIB | ENCSR019SQX | ENCSR087PFU |
| H3K4me1 | ENCSR493NBY | ENCSR271TFS | ENCSR831JSP |
| H3K27ac | ENCSR752UOD | ENCSR880SUY | ENCSR002YRE |
| H3K36me3 | ENCSR610IYQ | ENCSR476KTK | ENCSR437ORF |
| H3K79me2 | ENCSR059ICA | ENCSR301HRV | ENCSR713QLX |
| H3K9me3 | ENCSR999WH | ENCSR395USV | ENCSR055ZZY |
| H3K27me3 | ENCSR761DLU | ENCSR186OBR | ENCSR431UUY |
| MNase-seq | GSM1238700 | GSM1194220 | GSE21823 |
| RNA-seq | ENCSR000CTO | ENCSR000COU | ENCSR000CTQ |

**Table S2**. Number of unique mapped reads in each ChIP-seq and MNase-seq. Total ChIP-seq reads: 900,393,634, MNase-seq: 914,364,158.

|  | MCF7 | H1 | IMR90 |
| --- | --- | --- | --- |
| H3K4me3 | 44531458 | 16017110 | 25090195 |
| H3K4me1 | 103025509 | 20447461 | 43761941 |
| H3K27ac | 83658189 | 21915092 | 35468648 |
| H3K36me3 | 67798175 | 25246788 | 29178844 |
| H3K79me2 | 55463554 | 36725732 | 31582737 |
| H3K9me3 | 72984337 | 18931010 | 49736559 |
| H3K27me3 | 60563300 | 17579265 | 40687730 |
| Total | 488024522 | 156862458 | 255506654 |
| MNase-seq | 166146988 | 632768390 | 115448780 |

**Table S3**. The prior probability of the start state for the selected HMM.

| S1 | 1.01E-06 |
| --- | --- |
| S2 | 0.0625024 |
| S3 | 1.00E-06 |
| S4 | 1.01E-06 |
| S5 | 0.0437909 |
| S6 | 1.00E-06 |
| S7 | 1.00E-06 |
| S8 | 0.851992 |
| S9 | 1.03E-06 |
| S10 | 1.00E-06 |
| S11 | 1.00E-06 |
| S12 | 4.41E-06 |
| S13 | 0.0417024 |

**Table S4. BIC scores of the models with initial 21 to 25 states.**

|  | 1 | 2 | 3 | 4 | 5 |
| --- | --- | --- | --- | --- | --- |
| 21 | 6.07E+07 | 5.05E+07 | 6.05E+07 | 5.16E+07 | 5.28E+07 |
| 22 | 5.35E+07 | 5.05E+07 | 5.49E+07 | 5.58E+07 | 5.31E+07 |
| 23 | 4.92E+07 | 4.99E+07 | 5.22E+07 | 5.19E+07 | 5.19E+07 |
| 24 | 5.12E+07 | 5.09E+07 | 5.02E+07 | 5.13E+07 | 5.81E+07 |
| 25 | 5.29E+07 | 5.12E+07 | 5.87E+07 | 4.99E+07 | 5.36E+07 |

**Table S5**. Mark IDs of each histone mark.

| Mark | IDs |
| --- | --- |
| No Mark | 0 |
| H3K4me3 | 1 |
| H3K4me1 | 2 |
| H3K27ac | 4 |
| H3K36me3 | 8 |
| H3K79me2 | 16 |
| H3K9me3 | 32 |
| H3K27me3 | 64 |

**Table S6**. Common nucleosomes ratio in MCF7 cells.

| **Labels** | ChromHMM ratio | NucHMM ratio |
| --- | --- | --- |
| S2(C)_S9(N) | 63.79% | 98.63% |
| S3(C)_S4(N) | 82.18% | 87.37% |
| S4(C)_S5(N) | 85.02% | 97.10% |
| S5(C)_S10(N) | 98.67% | 99.48% |
| S7(C)_S2(N) | 98.97% | 70.94% |
| S9(C)_S3(N) | 93.76% | 98.84% |
| S10(C)_S11(N) | 95.76% | 97.64% |
| S11(C)_S7(N) | 77.76% | 66.09% |
| S12(C)_S13(N) | 95.03% | 84.03% |
| S13(C)_S1(N) | 94.00% | 93.19% |
| **Average ratio** | 88.49% | 89.33% |

**Table S7**. Common nucleosomes ratio in H1 cells.

| **Labels** | ChromHMM ratio | NucHMM ratio |
| --- | --- | --- |
| S2(C)_S9(N) | 38.68% | 97.91% |
| S3(C)_S4(N) | 88.50% | 91.21% |
| S4(C)_S5(N) | 86.62% | 98.46% |
| S5(C)_S10(N) | 97.08% | 96.15% |
| S7(C)_S2(N) | 98.76% | 83.14% |
| S9(C)_S3(N) | 91.34% | 99.21% |
| S10(C)_S11(N) | 93.35% | 97.93% |
| S11(C)_S7(N) | 78.18% | 51.88% |
| S12(C)_S13(N) | 92.57% | 93.81% |
| S13(C)_S1(N) | 92.92% | 94.13% |
| **Average ratio** | 85.80% | 90.38% |

**Table S8**. Common nucleosomes ratio in IMR90 cells.

| **Labels** | ChromHMM ratio | NucHMM ratio |
| --- | --- | --- |
| S2(C)_S9(N) | 40.53% | 95.07% |
| S3(C)_S4(N) | 77.59% | 82.90% |
| S4(C)_S5(N) | 81.11% | 88.53% |
| S5(C)_S10(N) | 97.63% | 99.22% |
| S7(C)_S2(N) | 98.69% | 86.76% |
| S9(C)_S3(N) | 91.74% | 98.04% |
| S10(C)_S11(N) | 92.12% | 96.55% |
| S11(C)_S7(N) | 85.45% | 57.25% |
| S12(C)_S13(N) | 92.66% | 93.54% |
| S13(C)_S1(N) | 87.46% | 92.17% |
| **Average ratio** | 84.50% | 89.00% |
