# Supplementary material for: NucHMM: a method for quantitative modeling of nucleosome organization identifying functional nucleosome states distinctly associated with splicing potentiality
Source: Genome Biol. 2021 Aug 26;22:250. doi: 10.1186/s13059-021-02465-1 (PMC8390234; doi:10.1186/s13059-021-02465-1)
Supplement: Supplementary file 5 — Additional file 5. Review history. [file 13059_2021_2465_MOESM5_ESM.docx]

Review History

**First round of review**

**Reviewer 1**

**Were you able to assess all statistics in the manuscript, including the appropriateness of statistical tests used? No**

**Were you able to directly test the methods? No**

**Comments to author:**

The authors describe NucHMM, an HMM-based model that utilizes MNase-seq and histone mark ChIP-seq to locate and categorize nucleosomes into states. They first identify nucleosome locations from MNase-seq data using iNPS. Separately, they call peaks from the different histone mark ChIP-seq datasets. The nucleosome positions are then binned using a bin-size of ~150bp and the histone marks overlapping the bins are somehow converted into observed symbols for the HMM; the details on the overlaps and the conversion are a little vague, but the result is a bit string indicating which histone marks are present in the bin, forming the observation sequence for NucHMM. The authors trained the HMM using 15-20 hidden states and chose the best model (20 hidden states) according to the smallest BIC score. They subsequently filter the hidden states down to 13, and retrain the model. They then apply the model by performing Viterbi decoding, after which the learned hidden states are assigned further functional annotations in the form of nucleosome positioning, phasing, spacing, and genomic locations. The paper describes the differences among these nucleosome states. It also identifies the enrichment of pioneer factors in the different states, as well as the states associated with "skipping exons".

Methods like NucHMM aim to address the important challenge of determining the arrangement of nucleosomes with different histone marks within the chromatin landscape. However, the current version of the manuscript fails to clearly explain many aspects of the model, as well as the calculation of various functional annotations, which is the central aspect of what makes this work interesting in the first place. The manuscript requires significantly improved writing to clarify the presentation. It also needs to provide more details and more justification for the large number of ad hoc parameters, filtering steps, and annotation expressions (Eq 9 is particularly impenetrable). In addition, the following comments need to be addressed to improve the manuscript.

Major comments:

1. The paper doesn't specifically define what each position in the HMM corresponds to. It seems that each position corresponds to a bin centered on a nucleosomal location. Assuming this is true, the observation sequence being modeled in these bins is not necessarily from contiguous segments of the genome. What is the maximum genomic distance allowed between two consecutive bins? How do you handle large gaps between bins where they occur? Also, is it reasonable to disregard the non-nucleosomal segments of the genome, considering that these segments contain important cis-regulatory elements that drive nucleosome positioning (or phasing, spacing, modifications, etc.) that in turn will affect gene regulation? It would be useful either to incorporate this knowledge into the model, or to address how the gaps and variable spacing between bins is accounted for.

2. Model selection with BIC is performed across 15-20 states, and 20 states are found to yield the best model, which is at the upper end of that range. How was that range selected in the first place? Given that the best model is at the end of the range, the authors should consider BIC scores for HMMs with even more states to demonstrate that the best model is indeed one with 20 states.

3. Related, it is hard to understand the rationale behind removing "redundant states". Why even do the BIC analysis to determine the optimal number of states if you then throw away states that emerge from that analysis? Also, how does the BIC score change after removing the redundant states? Do the redundant states merge with any specific hidden state that is retained? Do they correspond to any specific regions of the genome? Are they really "redundant" or are they merely "infrequent"?

4. The analyses are performed in aggregate across many loci, rather than providing some insights at specific loci. Fig. 4D is an exception, and shows one specific locus, but without the histone marks or gene relevance. It's hard to know if the state labels are reasonable, or even the functional annotation values. The authors should provide more details in this plot, and should include other locus plots like it.

5. The paper uses NucHMM to learn nucleosome states in three different cell types. It would be interesting to compare the nucleosome states across the three cell types for some specific genomic loci.

6. Better explanations of the calculations of nucleosome positioning and phasing are needed. The authors should also compare their results of nucleosome positioning and phasing with other existing methods, such as nucleR and Nucleosome Dynamics.

Minor comments:

7. In Table 1 the authors list the histone marks corresponding to each state. Some states are associated with more than one histone mark. Are there any specific combinations of histone marks that are more prevalent for these states?

8. Pg 13 line 50: Probability of calculation of mark-probability in Eq. 1: the values don't add up to 1 — is that reasonable? Are the values used anywhere aside from determining the histone mark enrichment for each state?

9. Pg 13 line 10: How much of overlap the nucleosome bins is allowed with each histone peak?

10. Figs 2E and 2F: also show heatmaps for better visualization.

11. Pg 5 lines 45-52: How long are the nucleosome arrays for each state? What is the average number of nucleosomes in each array?

12. Pg 5 lines 50-58: Why are the nucleosomes filtered out?

13. Pg 8 lines 56-57: Citation needed.

14. Define pioneer capacity. Calculation of pioneer capacity of TFs under Methods is hard to follow. Compare findings of pioneer capacity of TFs with literature evidence, if any.

15. Pg 11 line 12: where is the cutoff of 0.3 used?

16. When analyzing the pioneer factors and SEs, is it necessary to filter out the required states? It would be interesting to see that when the analysis is performed on all states, the states that come out as being important are the ones that are not currently being filtered out.

17. Fig S3: The comparison of the states of ChromHMM and NucHMM is hard to follow. A river plot could be useful in comparing the states of ChromHMM, NucHMM, and the histone marks.

**Reviewer 2**

**Were you able to assess all statistics in the manuscript, including the appropriateness of statistical tests used? Yes**

**Were you able to directly test the methods? No**

**Comments to author:**

The authors present NucHMM, a method for analyzing nuclosome positioning data (MNase-seq) in combination with other epigenetic data sets such as histone modifications. NucHMM is an adaptation of previously-developed segmentation and genome annotation (SAGA) methods such as Segway and ChromHMM. It takes as input a collection of these epigenomic data sets. It partitions the genome and assigns one of 11 state labels to each segment, such that each state label putatively annotations genomic regions with similar epigenetic activity. The authors analyze these annotations relative to other data sets and report a number of associations.

The interplay of histone modification, nucleosome positioning and gene expression is of great interest, and the method and results seem reasonable. However, the manuscript suffers from two major issues: novelty and presentation.

Novelty:

To my understanding, the main contributions of this manuscript relative to previous literature are:

1) The use of genomic bins based on nucleosome positioning. Previous SAGA methods use pre-defined bins of around 200bp each, while NucHMM defines bins based on nucleosome positions defined by MNase data.

2) Analysis relative to MNase-seq data. The authors relate each reported nucleosome state to nucleosome occupancy, which is relatively absent from previous chromatin state annotation analysis.

Point (1), the use of nucleosome bins, seems like a relatively minor improvement. I think there is the potential for novelty in point (2), but it was unclear what insights were learned based on the analysis relative to nucleosome positioning. Which nucleosome states have not been identified by other SAGA methods? Of the previously-identified nucleosome states, which associations with other data sets (MNase, TF ChIP-seq) are novel? Also, what is the advantage of using nucleosome-based bins rather than simply using 1bp bins, as used by, e.g., Segway?

Presentation: There are many issues in the text, such that the manuscript is quite hard to follow in the current form. In particular, I wasn't able to make sense of the nucleosome data processing section P14-15. Many terms are undefined, such as x, w, N, M. Several other examples are listed below.

Minor notes:

The authors should cite the full literature of related SAGA methods, such as what is listed in this review: https://arxiv.org/abs/2101.00688

On P12, the authors define a joint bit vector that maps each combination of 7 marks into one alphabet of 2^7 possibilities. Is this used differently than 7 binary values? Does the model use 7 Bernoulli distributions or one multinoulli with 128 options? I could not figure this out from the text.

P6L41: What does "our HMM is capable of capturing the chromatin states with the improved nucleosome level" mean?

It would help to use mnemonics for each state in the text to avoid frequent reference to Table 1.

Fig 2F: What is causing the large jump in state frequency at TTSs? Similar plots of the underlying epigenomic data usually show a smooth transitions around annotated TTSs; therefore, output of similar methods (e.g. Segway or ChromHMM) also usually show smooth transitions. If NucHMM can precisely identify this boundary, that would be a significant advantage. On the other hand, the authors should make sure the transition is not an artifact of aggregating different-length genes in the same plot.

Typos/text issues:

First sentence of abstract: "Currently, there is no computational method that can determine functional nucleosome states by integrating the nucleosome-level Hidden Markov model and quantitatively modeling nucleosome organization." Since "functional nucleosome states" and "quantitatively modeling nucleosome organization" are poorly defined, it is difficult to understand this sentence.

Hard to understand:

- P3L11

- P3L16

- P3L44

Frequent incorrect or missing articles ("a"/"the"). E.g. P3L7: "The nucleosome" or "a nucleosome".

P5L36: "resulted" -> "resulting"

**Authors Response**

**Point-by-point responses to the reviewers’ comments:**

***Response to Reviewer #1***

*Major comments:*

*1. The paper doesn't specifically define what each position in the HMM corresponds to. It seems that each position corresponds to a bin centered on a nucleosomal location. Assuming this is true, the observation sequence being modeled in these bins is not necessarily from contiguous segments of the genome. What is the maximum genomic distance allowed between two consecutive bins? How do you handle large gaps between bins where they occur? Also, is it reasonable to disregard the non-nucleosomal segments of the genome, considering that these segments contain important cis-regulatory elements that drive nucleosome positioning (or phasing, spacing, modifications, etc.) that in turn will affect gene regulation? It would be useful either to incorporate this knowledge into the model, or to address how the gaps and variable spacing between bins is accounted for.*

**Response**: The reviewer is right that each HMM observation corresponds to a bin centered on a nucleosomal location and the observation sequence is not contiguous segments of the genome. In the HMM training module, we did not set the threshold for the maximum genomic distance. Practically, the median of the genomic distance between two bins is 185 bp, 170 bp, 170 bp for MCF7, H1, and IMR90, respectively. The reasons for excluding the gaps between two bins in the HMM training are the following: 1) as histone modifications occur on the histone tails, using the bin centered on the nucleosome dyad is reasonable and able to capture all combinations of histone modifications; 2) Excluding gaps can save computational resources as its initiation matrix is half-sized compared to using the whole genome ‘bins’ (gap and nucleosomes). However, the gaps between two bins were modeled in the functional module. In this module, the nucleosome organization features, including positioning, spacing and phasing, were all included in the modeling and further used to define the nucleosome states from the HMM states. In NucHMM, we provided a parameter (nuchmm-init -- gap) allowing the users to keep the gap in the HMM training and a parameter (nuchmm-screen --cutoffdist) setting the threshold as the maximum genomic distance for identifying nucleosomal array. In addition, we have now added a new parameter (nuchmm-init --ciselements) in the program to accept cis-element chip-seq data files for the following HMM model training. And we trained a toy example to test the codes’ performance and showed the mark-state matrix below, demonstrating that NucHMM is able to train ChIP-seq of TFs data and Gap. However, in this paper, we would like to focus on providing a method that is able to define nucleosome states from the integrated histone modifications and nucleosome organization.

*2. Model selection with BIC is performed across 15-20 states, and 20 states are found to yield the best model, which is at the upper end of that range. How was that range selected in the first place? Given that the best model is at the end of the range, the authors should consider BIC scores for HMMs with even more states to demonstrate that the best model is indeed one with 20 states.*

**Response**: We selected 15-20 states as initial training states number based on our experience in the previous paper (Ref. 67). And we trained additional 25 HMM models from 21 to 25 states. The BIC scores are showed in the following table (**Suppl. Table S4**). From the table, we could prove that the model we used in the paper was the optimal model in all 55 HMM models from 15 – 25 initial state numbers.

|  | 1 | 2 | 3 | 4 | 5 |
| --- | --- | --- | --- | --- | --- |
| 21 | 6.07E+07 | 5.05E+07 | 6.05E+07 | 5.16E+07 | 5.28E+07 |
| 22 | 5.35E+07 | 5.05E+07 | 5.49E+07 | 5.58E+07 | 5.31E+07 |
| 23 | 4.92E+07 | 4.99E+07 | 5.22E+07 | 5.19E+07 | 5.19E+07 |
| 24 | 5.12E+07 | 5.09E+07 | 5.02E+07 | 5.13E+07 | 5.81E+07 |
| 25 | 5.29E+07 | 5.12E+07 | 5.87E+07 | 4.99E+07 | 5.36E+07 |

*3. Related, it is hard to understand the rationale behind removing "redundant states". Why even do the BIC analysis to determine the optimal number of states if you then throw away states that emerge from that analysis? Also, how does the BIC score change after removing the redundant states? Do the redundant states merge with any specific hidden state that is retained? Do they correspond to any specific regions of the genome? Are they really "redundant" or are they merely "infrequent"?*

**Response**: From our experience, the BIC score was used to select the optimal HMM model instead of selecting the optimal number of states. As BIC scores were shown in Fig. 2A, the BIC score varies within the same number of initial states. Importantly, as you mentioned, we compared the BIC scores and found that by removing the “redundant states” it decreased the BIC score and thus improved the HMM model. The BIC changing plot is shown below (**Suppl. Fig. S2A**).

As shown in Fig. 2B, S3/9/12/15/16/19/20 were removed. We plotted the state-mark matrix (**Suppl. Fig. S2C**) from the first HMM training. This matrix shown that S3/9/12/15/16/19/20 were not associated with any histone modifications. Besides, from the Sankey plot (**Suppl. Fig. S2D**), 99.995% (220,258 out of 220,271) “redundant states” were merged into S8/12 (background states) in the 2^nd^ HMM training, only with 3 nucleosomes from S3 to S9 and 10 nucleosome from S9 to S9. All in all, we concluded that the removed states are both infrequent and redundant to the model.

*4. The analyses are performed in aggregate across many loci, rather than providing some insights at specific loci. Fig. 4D is an exception, and shows one specific locus, but without the histone marks or gene relevance. It's hard to know if the state labels are reasonable, or even the functional annotation values. The authors should provide more details in this plot, and should include other locus plots like it.*

**Response**: In **Fig. 4D**, we wanted to demonstrate that our method for calculating nucleosome positioning score accurately captures the nucleosome positioning information in the MNase-seq data, so we didn’t contain histone marks tracks in this figure. To avoid misleading, we removed the state annotation track from **Fig. 4D**. However, it is indeed important to show the comprehensive tracks including histone marks, gene relevance and nucleosome states annotation. We thus provided such tracks with each of the nucleosome states in **Suppl. Fig. S15**.

*5. The paper uses NucHMM to learn nucleosome states in three different cell types. It would be interesting to compare the nucleosome states across the three cell types for some specific genomic loci.*

**Response**: We learned the nucleosome states jointly by creating a virtual concatenation of all chromosomes from all three cell types; and used the decoding algorithm (Viterbi) to call the cell type-specific state on each nucleosome on the basis of the patterns of histone marks and their spatial relationship in the cell type. Thus, every one of assigned states for a nucleosome is already cell type-specific. We here provided three examples in screenshots, FOXA1, ELL3, and POU5F1,
to show their cell type-specific nucleosome states.

*6. Better explanations of the calculations of nucleosome positioning and phasing are needed. The authors should also compare their results of nucleosome positioning and phasing with other existing methods, such as nucleR and Nucleosome Dynamics.*

**Response**: We have revised the Methods for the purpose of a better explanation. We compared our method with Nucleosome Dynamics (nucleR is included). We noticed that Nucleosome Dynamics assigned multiple nucleosomes to a wide range signal, while the underneath nucleosome calling program of NucHMM, iNPS, annotated this wide region as a single nucleosome. Thus, in the following comparison, we excluded those one-to-multiple mapping nucleosomes.

We first investigated the correlation of score_width and score_height between Nucleosome Dynamics and NucHMM. As there is no score_width and score_height output in the iNPS, we thus considered sqrt(1/width*pval-valley) as score_width and sqrt(height*pval-peak) as score_height for comparison purpose. We found that there is relatively high correlations between two programs: $r_{score\_width}=0.85, 0.82,0.83$; $r_{score\_height}=0.94, 0.95, 0.95$ for MCF7, H1, IMR90, respectively (**Suppl. Fig. S13A**). However, the positioning scores between the two programs show a relatively poor correlation $r_{positioning\_score}= 0.60, 0.69, 0.72$(**Suppl. Fig. S13B**). The potential reason would be that NucHMM defined the positioning score with the geometric-mean but Nucleosome Dynamics defined the positioning score with the arithmetic-mean. We then calculated the positioning score in the arithmetic-mean way to verify our guess and it indeed showed a more strong correlation $r= 0.88, 0.87, 0.85$, for MCF7, H1, IMR90, between the two programs (**Suppl. Fig. S13C**). In NucHMM, we would like to keep the geometric-mean in order to provide a bigger difference ($\mathrm{mean}_{arithmetic} \geq{mean}_{geometric}$) for the well positioned nucleosome and the poorly positioned nucleosome. For example, a well-positioned nucleosome with score_width = 0.9 and score_height = 0.9 would have a positioning score 0.9 for both geometric mean and arithmetic mean, but for a poorly positioned nucleosome with score_width = 0.1 and score_height =0.9, the geometric mean = 0.3 while the arithmetic mean = 0.5. We also attached some screenshots from the MuGVRE (Ref. 75) and showed the positioning score correlated well between NucHMM and Nucleosome Dynamics for well-positioned nucleosomes but had some differences between poorly-positioned nucleosomes (**Suppl. Fig. S14**).

For the phasing calculation, we found it is very hard to perform the comparison since the NucHMM calculates the phasing score based on the averaged nucleosome state array signal while Nucleosome Dynamics focus on the periodicity of the specific genes. But the Welch’s method used in NucHMM could be mathematically inter-changeable with the auto-correlation method used in Nucleosome Dynamics by the inversing Fourier transform of the power spectrum density. Thus, we believe the phasing score used in NucHMM captured the periodicity information of the nucleosome states array (**Suppl. Fig. S11**).

*Minor comments:*

*7. In Table 1 the authors list the histone marks corresponding to each state. Some states are associated with more than one histone mark. Are there any specific combinations of histone marks that are more prevalent for these states?*

**Response**: The histone marks listed in **Table 1** were derived from **Fig. 2D** with cutoff 0.3 (The answer for the 15^th^ comment). For those states having multiple marks, the combination of histone marks is more prevalent for those histone marks with higher possibility. For example, for S13 with K4me3/K4me1/K27ac/K79me2, K4me1/K79me2 are more prevalent than K4me3/K27ac.

*8. Pg 13 line 50: Probability of calculation of mark-probability in Eq. 1: the values don't add up to 1 — is that reasonable? Are the values used anywhere aside from determining the histone mark enrichment for each state?*

**Response**: Yes, all emission possibilities for a single state of HMM emission matrix should add up to 1. For the programs which use multivariate HMM (the observation is multi-dimensional histone mark features), e.g., chromHMM, the emission matrix is the mark-state matrix. Thus, the values add up to 1 in its’ mark-state matrix. However, NucHMM applied univariate HMM by digiting the combinations of histone marks first and converting the emission matrix back to the mark-state matrix by using the Eq.1. In our emission matrix (**Suppl. Fig. S3**), all values are added up to 1 for a state.

*9. Pg 13 line 10: How much of overlap the nucleosome bins is allowed with each histone peak?*

**Response**: In this paper, we used 30% overlapping as the cutoff. For example, for a 150 bp nucleosome bin, if a histone mark peak have 45 (150 * 0.3) bp overlapping with this nucleosome, we considered this nucleosome bin associated with the peak. In the program, we provided “--intersect_cutoff (default 0.3”) parameter in nuchmm-init subcommand to allow users to fine-tune the cutoff.

*10. Figs 2E and 2F: also show heatmaps for better visualization.*

**Response**: We have plotted the heatmaps (**Supple. Fig. S8**) for better visualization.

*11.* *Pg 5 lines 45-52: How long are the nucleosome arrays for each state? What is the average number of nucleosomes in each array?*

**Response**: The length of the nucleosome array for each state varies among the genomic loci and the ‘Ave. No. of. Nucleosomes’ column in Table 1 shows the average number of nucleosomes in each array. The length range of the nucleosome array for each nucleosome state is in the table below.

| Functional nucleosome states (HMM states) | Ave. No. of Nucleosomes | No. Range of Nucleosomes |
| --- | --- | --- |
| NucS1 (S1) | 3.99 | 2~18 |
| NucS2 (S2) | 8.49 | 3~38 |
| NucS3 (S3) | 5.82 | 2~29 |
| NucS4 (S4) | 4.13 | 2~16 |
| NucS5 (S5) | 4.56 | 1~6 |
| NucS6 (S6) | 3.74 | 2~15 |
| NucS7 (S7) | 5.32 | 2~26 |
| NucS8 (S9) | 2.09 | 1~6 |
| NucS9 (S10) | 7.37 | 3~32 |
| NucS10 (S11) | 4.55 | 2~21 |
| NucS11 (S13) | 4.67 | 2~19 |

*12.* *Pg 5 lines 50-58: Why are the nucleosomes filtered out?*

**Response**: There are several reasons for filtering out some nucleosomes: 1) since we wanted to identify functional nucleosome states, the nucleosomes without any histone marks were filtered. 2) as we profile nucleosome organization by averaging the signal of all nucleosome states’ array, we also wanted to filter out some ‘outliers’. For instance, if a nucleosome is not in a nucleosome array or is extremely fuzzy, we would filter it out; and if a nucleosome at the start/end of the array does not fit the positioning, phasing and spacing score of the nucleosome state of this array, we would drop it.

*13.* *Pg 8 lines 56-57: Citation needed.*

**Response**: We added the citations for lines 56-57: Refs 73, 74.

*14.* *Define pioneer capacity. Calculation of pioneer capacity of TFs under Methods is hard to follow. Compare findings of pioneer capacity of TFs with literature evidence, if any.*

**Response**: Pioneer capacity is the capability of a transcription factor that directly binds to condensed chromatin and opens the chromatin. In another word, it is the measurement of how possible a transcription factor could be a pioneer factor. For calculating the pioneer capacity, generally, we first generated two matrices: a matrix profiles the correlations between TFs and NucSs (TNS matrix) and a matrix profiles the correlation between TFs (TR matrix). Then, we got three measurements from these two matrices: 1. The measurement of the chromatin openness associated with a TF (measured from the TNS matrix). For a pioneer factor, this measurement should be large and show its ability to open the chromatin. 2. The measurement of TF-TF affinity, which told us how ‘attractive’ of a TF to another TFs. A pioneer factor should have the ability to recruit other TFs (measured from the TR matrix). 3. The measurement of enrichment changes of a TF between low expressed genes and high expressed genes in the heterochromatin associated NucS as well as in the poised NucS (measured from the TNS matrix). Suppose a TF is more enriched in low expressed genes than high expressed genes in the heterochromatin associated NucS and is more enriched in high expressed genes in the poised NucS, we would give it a higher score for this measurement. The more detailed steps and the equation are demonstrated in the Methods (Pages 16-17). We have listed several papers in the discussion and here we provided additional related papers.

a) GATA3; Tanaka, Hiroki, et al. "Interaction of the pioneer transcription factor GATA3 with nucleosomes." Nature communications 11.1 (2020): 1-10
b) EGR1; Trizzino, Marco, et al. "EGR1 is a gatekeeper of inflammatory enhancers in human macrophages." Science Advances 7.3 (2021): eaaz8836.
c) FOS; Vierbuchen, Thomas, et al. "AP-1 transcription factors and the BAF complex mediate signal-dependent enhancer selection." Molecular cell 68.6 (2017): 1067-1082.

*15.* *Pg 11 line 12: where is the cutoff of 0.3 used?*

**Response**: We rewrote the Pg 11 line 12 for better understanding. The 0.3 cutoff was used to determine any specific histone mark to be associated with a nucleosome state.

*16.* *When analyzing the pioneer factors and SEs, is it necessary to filter out the required states? It would be interesting to see that when the analysis is performed on all states, the states that come out as being important are the ones that are not currently being filtered out.*

**Response**: We thought it’d better to filter out the required states because the non-relevant NucSs had provided little information to the meaningful biological results. For example, for the nucleosome state that mainly located in the promoter and associated with active histone marks, e.g. NucS8, it’s already in an ‘opened’ environment and thus have a small chance in being involved in opening chromatin activities of the pioneer factors. For analyzing the pioneer factors, only 4 states are associated with distal region, we excluded NucS2 based on our current knowledge (Refs 73, 74). For analyzing the SEs, our previous finding (Ye et al. "Computational analysis reveals a correlation of exon-skipping events with splicing, transcription and epigenetic factors." 2014) showed that although H3K36me3 was enriched in the gene body region, it wasn’t related with SE events. Here we also confirmed that NucSs with H3K36me3 is evenly or more enriched in background exons than exons involved in exon skipping in MCF7, H1 and IMR90 respectively (above figure). Thus, we only used the NucSs mainly enriched with H3K79me2 in the SEs analysis.

*17.* *Fig S3: The comparison of the states of ChromHMM and NucHMM is hard to follow. A river plot could be useful in comparing the states of ChromHMM, NucHMM, and the histone marks.*

**Response**: We have plotted the river plot (**Suppl. Fig. S5E**) for better visualization of the states switch between ChromHMM and NucHMM.

***_____________________________***

***Response to Reviewer #2***

*Major comments:*

*1. Point (1), the use of nucleosome bins, seems like a relatively minor improvement. I think there is the potential for novelty in point (2), but it was unclear what insights were learned based on the analysis relative to nucleosome positioning. Which nucleosome states have not been identified by other SAGA methods? Of the previously-identified nucleosome states, which associations with other data sets (MNase, TF ChIP-seq) are novel? Also, what is the advantage of using nucleosome-based bins rather than simply using 1bp bins, as used by, e.g., Segway?*

**Response**: We would like to point out that the functional nucleosome states we defined in this paper are resulted from integrating the quantified features of nucleosome organization and genomic regions with trained HMM states based on the nucleosome bins. We performed a series of functional filtering in the functioning module, including: a) filtering out the ‘stand-alone’ nucleosomes assigned to a nucleosome state within a long nucleosome array; b) filtering out nucleosomes with extremely low positioning scores; and c) filtering out nucleosomes in a unphased situation at the beginning or the end. The following screenshots showed the filtering cases. By performing the functioning module, we identified the nucleosome states based on both the nucleosome-bin based HMM states and nucleosome organization features. Therefore, we felt that all of the nucleosome states identified by NucHMM are novel.

As for what insights we learned based on the analysis relative to nucleosome positioning, we feel that our identification of 11 functional nucleosome states (Table 1) and computational analyses offer a mechanistic insight into the interplay among nucleosome organization (including nucleosome positioning), pioneer factors and splicing process. For example, as shown in Table 1, the nucleosome positioning of the compacting nucleosome states (long nucleosome array) with relatively high nucleosome positioning, such as NucS2 (K9me3) and NucS9 (K27me3), might create a spatial barrier for transcription factors to bind their motif. The transcription related nucleosome state, NucS8, has a relatively low nucleosome positioning, indicating that the nucleosomes at the promoter experiences frequently dynamic changes such as assembly/disassembly, switches and shift processes.

We applied Segway to train the data. As we didn’t find Segway’s command that allows to train all three cell types together, we trained the data in the individual cell type respectively. We found there are several differences between NucHMM and Segway. Firstly, as Segway run with 1bp resolution and has to run cell type individually, NucHMM would train the model with a little bit more efficiency. The training time of NucHMM for the current dataset was ~13h for a single model of all cell types, while Segway needed ~16h*3 with --minibatch-fraction=0.01 and ~50h*3 with –minibatch-fraction=0.1 on our server, and we assumed that the training time for the entire genome would be much longer. Secondly, similar to the comparison between NucHMM and ChromHMM, we found 1223,73, 132,244, 156,709 nucleosomes (**Suppl. Fig. S6A**) identified with two states by Segway in MCF7, H1 and IMR90 respectively. The screenshots shows (**Suppl. Fig. S6B**) these bi-annotated cases. Further, NucHMM has a little bit larger states change per kb 0.23, 0.22, 0.32 than Segway 0.14, 0.15, 0.20 in MCF7, H1 and IMR90. Finally, in the 13-states DBN model, we found that the states identified by Segway are tended to have more high-probability marks than NucHMM, which made a little bit more difficult for users to tell the difference between the states.

However, we recognized that the model from Segway might not be the optimal one that Segway might be able to learn since many parameters in Segway could be fine-tuned. For example, 1) we only used one initial seed to train DBN (not set --num-instances), which only represents a local-optimal network for the data; 2) we only used the minibatch parameter (0.1) to train the network to speed up the training process, which might not reflect the optimal result that trained by the entire genome; 3) for the purpose of the comparison, we used the graph with 13 nodes which might not be the optimal graph network nodes; and 4) we only applied the default parameters to train the network although Segway has many other parameters for fine-tuning.

*2. There are many issues in the text, such that the manuscript is quite hard to follow in the current form. In particular, I wasn't able to make sense of the nucleosome data processing section P14-15. Many terms are undefined, such as x, w, N, M. Several other examples are listed below.*

**Response**: Thanks for pointing it out. We have re-wrote those paragraphs to make it clear for readers.

*Minor comments*

*3. The authors should cite the full literature of related SAGA methods, such as what is listed in this review: https://arxiv.org/abs/2101.00688*

**Response**: We cited this review paper (Ref. 38) and all related SAGA methods (Refs 38-68).

*4. On P12, the authors define a joint bit vector that maps each combination of 7 marks into one alphabet of 2^7 possibilities. Is this used differently than 7 binary values? Does the model use 7 Bernoulli distributions or one multinoulli with 128 options? I could not figure this out from the text.*

**Response**: Yes, it is a one multinouli with 128 options and is used differently than using 7 binary values since NucHMM used univariate HMM. In NucHMM, we chose to assign a single value to an observation. In this case, 7 marks have possible 2^7 combination values. For example, a nucleosome with H3K4me3H3/K27ac was assigned with the value 5 (b101) and H3K4me3/H3K4me1/H3K27ac was assigned with the value 7 (b111) in the NucHMM’s observation sequence.

*5. P6L41: What does "our HMM is capable of capturing the chromatin states with the improved nucleosome level" mean?*

**Response**: We found that there are more than 683k/411k nucleosomes that crossed the boundary of two HMM states (bi-annotated) identified by ChromHMM and Segway in all three cell types. Although ChromHMM could run bin with ~150bp and Segway could run with 1bp, they are not designed to deal with many nucleosomes locating among different states. The IGV screenshots attached in Comment 1 show the bi-annotated scenarios. While NucHMM trains the nucleosome state by binning on the nucleosome, we believe it can capture the chromatin states with the improved nucleosome level.

*6. It would help to use mnemonics for each state in the text to avoid frequent reference to Table 1.*

**Response**: We have clearly used mnemonics for each nucleosome state at the beginning of each paragraph to avoid frequently referring to **Table 1**.

*7. Fig 2F: What is causing the large jump in state frequency at TTSs? Similar plots of the underlying epigenomic data usually show a smooth transitions around annotated TTSs; therefore, output of similar methods (e.g. Segway or ChromHMM) also usually show smooth transitions. If NucHMM can precisely identify this boundary, that would be a significant advantage. On the other hand, the authors should make sure the transition is not an artifact of aggregating different-length genes in the same plot.*

**Response**: Thanks for pointing this out! It is an artifact resulted from aggregating different-length genes in the same plot. To avoid the misleading, we removed the 10kb downstream TTS region from the Fig. 2F and plotted an additional states distribution in the TTS region from -4k to 4k (**Suppl. Fig. S9**). From this TTS plot, we can see a clear ‘valley’ for the majority of the states, which is consistent with the evidence that nucleosomes are depleted in the TTSs (Teif, Vladimir B., et al. "Genome-wide nucleosome positioning during embryonic stem cell development." Nature structural & molecular biology 19.11 (2012): 1185)

*8. Typos/text issues: First sentence of abstract: "Currently, there is no computational method that can determine functional nucleosome states by integrating the nucleosome-level Hidden Markov model and quantitatively modeling nucleosome organization." Since "functional nucleosome states" and "quantitatively modeling nucleosome organization" are poorly defined, it is difficult to understand this sentence.*

**Response**: As the abstract has limited to 100 words, it is hard for us to provide the detailed definition in the abstract. But we defined the functional nucleosome state and quantitatively modeling nucleosome organization in the **Introduction**. We also provided the definition here:

1. Functional nucleosome state: a state encoding combinatorial histone marks and nucleosome organization features that performs a specific function and responds to the environment and intercellular signaling. For example, the composition of promoter nucleosomes facilitate or inhibit transcription (Jiang & Pugh, Nature Reviews Genetics, 2009, 10, 161-172. Hormone-induced exon inclusion correlates with higher nucleosome occupancy at the exon with higher RNA polymerase II accumulation (Iannone et al, RNA, 2015, 21:360-374.
2. Quantitatively modeling nucleosome organization: the measurements of the phasing of a nucleosome array, the spacing between two dyads of the nucleosomes, and the degree of nucleosome positioning in a quantitative manner.

*9. Hard to understand: P3L11, P3L16, P3L44*

**Response**: We have revised these sentences to make them more understandable.

*10. Frequent incorrect or missing articles ("a"/"the"). E.g. P3L7: "The nucleosome" or "a nucleosome". P5L36: "resulted" -> "resulting".*

**Response**: We have made these changes across the text as suggested.

**Second round of review**

**Reviewer 1**

The authors have addressed many of my concerns. The part of the paper that discusses the construction of the HMM is now quite thorough. However, I still have concerns regarding the calculations of both pioneer capacity of transcription factors and splicing potentiality of skipping exons, which are behind some of the potentially most interesting results. The authors have taken heuristic measures to construct the equations, which makes some of the choices hard to follow, justify, or interpret.

Major comments:

1. Eq. 6: What is Coef_rank? How is it adjusted to filter the threshold? What is the rationale behind the equation?

2. Eq. 9 is still very hard to follow. It seems that the authors made some assumptions about each of the nucleosomal states and, depending on the choices, used values corresponding to the different states in various parts of the equation. For example, why is the enrichment of a TF in a gene in state S9 divided by the same metric in states S4 and S5? The authors should extensively elaborate on this because this equation is both extremely ad hoc and extremely hard to understand or justify.

3. Similarly, the authors should provide a better explanation for Eq 10. Here, the authors have noted the meaning of each of the terms in the equation but not the reasoning behind the equation itself. It’s very hard to trust results when the transformations used to get those results cannot be understood clearly.

4. Previous Comment #5: This is not quite what I was asking for. It would be more convincing if the authors took the annotated nucleosomes from the three cell types and assessed the differences in the annotations across the three cell types. The analysis could be performed genome-wide and then the authors can filter the segments with the most differences. It would be interesting to see if these differences lie near specific genes.

Minor comments:

5. Previous Comment #8: My comment was regarding the calculation of the probability of individual histone marks in Eq. 1. Do they add up to 1? As in, if the probability of individual histone marks were calculated separately for all the histone marks, will the values add up to 1?

6. Previous Comment #10 and Supp Fig. S8: It is interesting to note that the distribution of state S10 is particularly different upstream of TSS in H1 cells. This brings me back to my previous comment (point #4 above): Which regions of the genome are annotated differently among the three cell types?

7. The supplementary section indicates that NucHMM could capture the states that are missed by ChromHMM if the length is adjusted. The authors need to demonstrate that this is indeed true. Moreover, what happens when ChromHMM is run with more than 13 states (15 or 17 states)? Will it capture the differences in states that are missed with 13 states of ChromHMM? Additionally, the mapping of states between ChromHMM and NucHMM seems to be incorrect in Supp. Fig. S4 as well as in Supplementary pg 3 (not visually right, and does not line up with what is shown in the river plot in Supp Fig S5E).

**Reviewer 2**

The authors addressed several of my concerns, including the relationship to 1-bp analysis. However, several concerns remain.

- My concerns regarding the novelty of the proposed approach remain. The relationship of NucHMM states to ChromHMM in Figs S4-5 show that the two models produce extremely similar results. It seems that the only state unique to NucHMM is their S6 (K4me1+K36me3). It is unclear whether several reported results are novel:

1) Has the K4me1+K36me3 pattern of NucS6 been observed before? How can we be sure that this idenfication is robust?

2) The authors show that Segway and ChromHMM annotations cross nucleosome boundaries, which seems like a big advantage of NucHMM. Can we measure the advantage given by this fact? For example, does NucHMM identify the location of TSSs more precisely than Segway and ChromHMM?

3) A number of studies, such as the following, investigate the relationship between nucleosome positioning and chromatin state. Which patterns reported here have been identified before?

Lorzadeh, A., Bilenky, M., Hammond, C., Knapp, D. J., Li, L., Miller, P. H., ... & Hirst, M. (2016). Nucleosome density ChIP-Seq identifies distinct chromatin modification signatures associated with MNase accessibility. Cell reports, 17(8), 2112-2124.

- S4: "There were two states that were uniquely identified by ChromHMM: S6(C) and S8(C), and one state that was uniquely identified by NucHMM: S6(N)." How did the authors determine which states are unique vs shared? Visual inspection or some specific criterion?

- I agree with R1's comment regarding the distribution of bin distances. In their response, the authors mentioned the median distance between the bins. They should show the full distribution -- are there some pairs with much higher gaps? What is the effect of these large gaps?

- The text has been substantially improved. However, some issues remain:

P14 paragraph starting on L12. I don't understand this paragraph. What does it mean to "average all of the nucleosome arrays of the certain nucleosome state"?

P13L34: run -> ran.

P14L17: Grammar issues.

P14L21: What is "it"?

**Authors Response**

**Point-by-point responses to the reviewers’ comments:**

***Response to Reviewer #1***

**Major comments:**

*1. Eq. 6: What is Coef_rank? How is it adjusted to filter the threshold? What is the rationale behind the equation?*

**Response:** Coef_rank_ is a parameter subjectively used to adjust the spacing range by users with 1 bp as the default. The bigger Coef_rank_ is, the wider spacing range is. For example, based on Eq 6, for a NucS8 (Rank 1) array, the 2^nd^ nucleosome is considered within the range if it is between [175 - 1*(5+1*1), 175 + 1*(5+1*1)] = [169, 181], but for a NucS10 (Rank 11) array, the 2^nd^ nucleosome is considered within the range if it is between [185 - 1*(5+11*1), 185 + 1*(5+11*1)] = [169, 201]. We also recognized that Coef_rank_ might be misleading the readers to think the coefficient varying with the ranks. We thus changed it to Coef_range_ in the Eq 6 to avoid further misunderstanding. We have further clarified Eq 6 in much details in Page 15.

The rationale behind the Eq 6: as demonstrated in **Fig. 3B**, we observed that the 1^st^ nucleosome in a NucS array is uniformly phased, and the following nucleosomes become increasingly fluctuating along the NucS array. Thus, it is essential to use a quantitative formula to reflect such fluctuation and to further define a spacing range for each of nucleosomes after the 2^nd^ nucleosome in the NucS array. As specified in Eq 6 for the meaning of each of the terms, the equation is carefully designed to reflect the fluctuation and thus appropriately measure the spacing range. As expected, the spacing ranges of the first few nucleosomes are smaller than the last few nucleosomes in a NucS array.

*2. Eq. 9 is still very hard to follow. It seems that the authors made some assumptions about each of the nucleosomal states and, depending on the choices, used values corresponding to the different states in various parts of the equation. For example, why is the enrichment of a TF in a gene in state S9 divided by the same metric in states S4 and S5? The authors should extensively elaborate on this because this equation is both extremely ad hoc and extremely hard to understand or justify.*

**Response:** We have rewrote the whole paragraph (Pages 16-19) and explained it in much details for each of Eq 9-15. The reason why the enrichment of a TF in a gene in NucS9 divided by the same metric in NucS4 and NucS5 is that since NucS9 has a larger nucleosome spacing than NucS4 and NucS5 do, we used the ratio of normalized TF counts of NucS9 to NucS4+NucS5 to measure the ability of a TF to open the chromatin, where this is reflected in the first part of the equation.

*3. Similarly, the authors should provide a better explanation for Eq 10. Here, the authors have noted the meaning of each of the terms in the equation but not the reasoning behind the equation itself.  It’s very hard to trust results when the transformations used to get those results cannot be understood clearly.*

**Response:** We have added more detailed explanations for Eq 16 (old Eq 10) in the **Methods** (Page 19). In Refs 30-37, the authors suggested that nucleosome organization and certain histone marks played an important role in the skipping exon event. Our previous studies (Refs 37 and 82) found H3K79me2 is enriched within SEs than other marks. Thus, we selected the H3K79me2 associated NucSs and measured their SE potentiality. The Fréchet Distance ($norm\left( frdist \right)$) is used to measure the difference of the averaged nucleosome array signal (containing nucleosome spacing and phasing information) between rSE’s exons and urSE’s exons (**Suppl. Fig. 19**). The $norm(abs({diff}_{nucpos})$ measured the difference of the nucleosome positioning between rSE and urSE group. The larger the Fréchet distance and nucleosome positioning implied a higher SE potentiality of the NucS. The last ${coef}_{event-counts}= norm(\frac{{number of NucS}_{rSE}}{number of NucS})$ is to measure the ‘abundance’ of the NucS in the rSE group.

*4. Previous Comment #5: This is not quite what I was asking for. It would be more convincing if the authors took the annotated nucleosomes from the three cell types and assessed the differences in the annotations across the three cell types. The analysis could be performed genome-wide and then the authors can filter the segments with the most differences. It would be interesting to see if these differences lie near specific genes.*

**Response**: We have performed cell type-specific NucSs-genes analysis (**Suppl. Note** and **Suppl. Fig. S14**). Briefly, we built a signal matrix with the NucS array signal in each of all refseq genes, where the rows represent genes and the columns represent the genomic region of the NucS. We calculated cell type A-specific signal matrix (such as MCF7) by its subtracting other cell types’ signal matrices (such as H1 and IMR90). We finally calculated the signal fold change and a p-value with an ANOVA test for each gene across three signal matrices. The lists of cell type-specific NuSs-genes were provided in **Additional files 2-4**.

**Minor comments:**

*5. Previous Comment #8: My comment was regarding the calculation of the probability of individual histone marks in Eq. 1. Do they add up to 1? As in, if the probability of individual histone marks were calculated separately for all the histone marks, will the values add up to 1?*

**Response:** As we had mentioned in the previous response, the probability of the emission matrix of the HMM (**Suppl. Fig. S3**) are added up to 1 for any particular state, *i.e.*, the sum of 128 observation columns equals to 1. The shape of the emission matrix is ${number}_{states}\times2^{{number}_{state}}$. The Eq 1 is used to calculate the probability of individual marks from those $2^{{number}_{state}}$ observations. Thus, the probability of histone marks didn’t add up to 1 in the **Fig. 2D**. For example, if observation 0b0001001 has probability 0.6 in the emission matrix, then this 0.6 will count into the probability for both H3K4me3 and H3K79me2 observations. The sum of probability of H3K4me3 and H3K79me2 is already larger than 1.

*6. Previous Comment #10 and Supp Fig. S8: It is interesting to note that the distribution of state S10 is particularly different upstream of TSS in H1 cells. This brings me back to my previous comment (point #4 above): Which regions of the genome are annotated differently among the three cell types?*

**Response:** We have further performed cell type-specific (or differential) NucSs-genes analysis based on the annotated NucS array (**Suppl. Note** and **Suppl. Fig. S14**). For each NucS, we found it has different sets of genes in each cell type (genes list are in the **Additional files 2-4**). Please note that each of 11 NucSs has already been annotated in a particular genomic region in which it is defined in the Functioning Module, so it is a different from the states directly learned from nucleosome-based HMM.

*7. The supplementary section indicates that NucHMM could capture the states that are missed by ChromHMM if the length is adjusted. The authors need to demonstrate that this is indeed true. Moreover, what happens when ChromHMM is run with more than 13 states (15 or 17 states)? Will it capture the differences in states that are missed with 13 states of ChromHMM?  Additionally, the mapping of states between ChromHMM and NucHMM seems to be incorrect in Supp. Fig. S4 as well as in Supplementary pg 3 (not visually right, and does not line up with what is shown in the river plot in Supp Fig S5E).*

**Response:** We visualized some loci for S6 trained from NucHMM to demonstrate that the state captured by NucHMM is indeed true (Screen shots #1-2).

**Screen shot #1:**

**Screen shot #2:**

We trained ChromHMM with 15 and 17 states respectively with each of the emission matrix shown below. The 15-states ChromHMM model showed similar states with the 13-states model, while 17-states model indeed captured some new states expanded from the old 13-states matrix.

However, when we visually inspected new states, for example E1 in 17-states model, it turned out many annotated E1’s are either 1) not associated with any nucleosomes (Screen shots #3, #4); or 2) even if associated with nucleosomes but over-annotated to the edge of the histone mark which might belong to another state (Screen shots #4, #5).

**Screen shot #3:**

**Screen shot #4:**

**Screen shot #5:**

We also corrected mismatch typos in **Suppl. Fig. S4** as well as in **Suppl. Notes** at Page 3.

*-------------------------------------------------------------------*

***Response to Reviewer #2***

*- My concerns regarding the novelty of the proposed approach remain. The relationship of NucHMM states to ChromHMM in Figs S4-5 show that the two models produce extremely similar results. It seems that the only state unique to NucHMM is their S6 (K4me1+K36me3). It is unclear whether several reported results are novel:*

**Response:** We should point out that the novelty of our NucHMM is not about how many more or less states it can get in comparison with other epigenetic annotation tools such as ChromHMM or Segway. We think the major novelties of our NucHMM lie in the following aspects: 1) we build nucleosome-based observations in the ‘Initialization’ module and used it for the univariate HMM training. This nucleosome-level observation allows us to precisely annotate combinatorial histone modification on the nucleosomes (**Suppl. Fig. S5A, S6A** and **Fig. S6C**); 2) we apply a ‘Functioning’ module to convert HMM states to functional nucleosome states (NucSs), which are associated with not only combinatorial histone modifications, but also nucleosome organization features, including nucleosome phasing, spacing and positioning; and 3) our nucleosome-based HMM is able to accurately capture the 5’TSS (**Suppl. Fig. S6B**). To the best of our knowledge, this is the first computational tool currently available that is able to genome-wide annotate functional nucleosome states.

*1) Has the K4me1+K36me3 pattern of NucS6 been observed before? How can we be sure that this identification is robust?*

**Response:** We found a few papers that have reported the K4me1 + K36me3 pattern which is usually an intronic enhancer.

*Soldi et al., "Chromatin proteomics reveals novel combinatorial histone modification signatures that mark distinct subpopulations of macrophage enhancers" Nucleic acids research, 45:12195-12213, 2017.*

*Siu et al., “Characterization of the human thyroid epigenome”, Journal of Endocrinology, 235: 153-165, 2017.*

*Liu et al., “Transcription factor-associated combinatorial epigenetic pattern reveals higher transcriptional activity of TCF7L2-regulated intragenic enhancers”, BMC Genomics, 18:375, 2017.*

*2) The authors show that Segway and ChromHMM annotations cross nucleosome boundaries, which seems like a big advantage of NucHMM. Can we measure the advantage given by this fact? For example, does NucHMM identify the location of TSSs more precisely than Segway and ChromHMM?*

**Response:** We plotted the distribution of promoter-associated states, NucS8 in NucHMM, E2 in ChromHMM, and Promoter in Segway respectively. We observed that there is a clear gap at 5’TSS in the NucS8/NucHMM, a minor gap in Promoter/Segway and no gap in E2/ChromHMM (attached figure). As expected, there should be a nucleosome free region (NFR) at 5’TSS. Therefore, our nucleosome-based NucHMM is able to accurately capture the 5’TSS.

 *3) A number of studies, such as the following, investigate the relationship between nucleosome positioning and chromatin state. Which patterns reported here have been identified before?*
 *Lorzadeh, A., Bilenky, M., Hammond, C., Knapp, D. J., Li, L., Miller, P. H., ... & Hirst, M. (2016). Nucleosome density ChIP-Seq identifies distinct chromatin modification signatures associated with MNase accessibility. Cell reports, 17(8), 2112-2124.*

**Response:** Thanks for providing this paper. The paper provides an insight into using histone modifications and MNase accessibility to define different types of promoters. However, this paper mainly focused on two histone modifications (K4me3 and K27me3) on the promoter region and didn’t provide any nucleosome positioning information though provided a fragment length distribution in Figure. S2. However, since the x-ticks was not well labeled, it is hard for us to get the accurate fragment length of the peak and to provide any meaningful comparison.

We found a paper (Valouev, Anton, et al. "Determinants of nucleosome organization in primary human cells." Nature 474.7352 (2011): 516-520), in which the authors have found that K4me1 has a relatively small nucleosome spacing of 178 bp and K9me3/K27me3 have a larger nucleosome spacing of 205 bp (Fig. 2b). These results are consistent with what we found in our study (Table 1). In addition, in Fig. 3d, the authors showed that H3K4me3 was associated with a good phasing property, which is also in line with our finding that NucS8 has the highest phasing score. As for nucleosome positioning, to the best of our knowledge, we didn’t find any papers that quantitatively examine the relationship between combinatorial histone modifications and nucleosome positioning. To test the reliability, we performed a comparison of nucleosome positioning between NucHMM and NucleR (**Suppl. Note, Suppl. Fig. S21-22**) and we think our results were able to accurately capture the nucleosome positioning.

*4): "There were two states that were uniquely identified by ChromHMM: S6(C) and S8(C), and one state that was uniquely identified by NucHMM: S6(N)." How did the authors determine which states are unique vs shared? Visual inspection or some specific criterion?*

**Response:** We first used state-mark matrix (**Suppl. Fig. S4**) to determine which states are unique or shared such that the state is shared between NucHMM and ChromHMM if it has the same combination of histone modifications, otherwise, the state is unique to either program. We then used its distribution related to the genomic location to double check if it distributes appropriately in its respective genomic region. We finally visually inspected some loci to further confirm the results.

*5) I agree with R1's comment regarding the distribution of bin distances. In their response, the authors mentioned the median distance between the bins. They should show the full distribution -- are there some pairs with much higher gaps? What is the effect of these large gaps?*

**Response:** We have plotted the full distributions for all three cell types (see the attached figure below). We do observe very small percentage of the pairs of nucleosomes with a higher gap larger than 512 (= 2^9^) bp. However, the larger gap doesn’t affect the ‘Initialization’ and ‘Training’ module as we only used observations on the nucleosomes to train HMM. But the larger gap will affect ‘Functioning’ module in terms of determining the nucleosome array. By default, if two nucleosomes have a gap larger than 350 bp (--cutoffdist), they will be assigned into different nucleosome arrays even if they belong to the same HMM state.

*6) P14 paragraph starting on L12. I don't understand this paragraph. What does it mean to "average all of the nucleosome arrays of the certain nucleosome state"?*

*P13L34: run -> ran.*

*P14L17: Grammar issues.*

*P14L21: What is "it"?*

**Response:** Thanks for the suggestions. We have corrected these errors in the text.

"average all of the nucleosome arrays of the certain nucleosome state" means that we summed all nucleosome array signals with a particular nucleosome state and then divided it by the number of the nucleosome arrays within this nucleosome state. To avoid further confusion, we slightly rephrased the sentences (Page 14).

‘it’ refers to the Gaussian smoothed signal in the previous sentence.

**Third round of Review**

**Reviewer 1**

The authors have added further explanations about the equations in the manuscript although the text continues to need significant improvement in clarity and fluency.

The authors define pioneer capacity of transcription factors (TFs) as the ability of the TFs to open up chromatin. They quantify this measure using the nucleosomal states learned by their model NucHMM and the ChIP-seq signal of the TFs. The main drawback of this choice of quantification is that it does not incorporate TF motifs into the calculation. Therefore, it is hard to find this pioneer capacity calculation reliable enough to provide useful biological insight.

Major comments:

1. In the TNS matrix, the ChIP-seq enrichment signal for a TF is aggregated across genes labelled as {U, M, L, H} and grouped by the nucleosomal states learned by NucHMM. The chromatin-opening ability for a TF is calculated by taking the ratio of its enrichment signal lying within state NucS9 versus lying within states NucS4 and NucS5. Given that no motif enrichment analysis is performed, it is hard to explain using only this ratio whether a TF actually has high chromatin-opening ability. For example, the computed ratio may be high only because the TF’s motif is often present within NucS9 segments of chosen genes but absent in NucS4 and NucS5 segments. This analysis needs to account for whether potential binding sites even exist, perhaps by retaining only those segments that have TF motifs when calculating pioneer capacity. Methods like FIMO can be used to first obtain a set of TF binding sites.

2. Eqn 10: Why is chromatin-opening ability reweighted again when the values are normalized in eqn 9?

3. Eqns 12-13 are still very hard to understand.

Minor comments:

4. Fig S19: This is a plot of averaged signal and not Fréchet distance. Fréchet distance is only shown for a small segment. Caption needs to be modified.

**Reviewer 2**

1) Regarding novelty: The description of novelty given in the reviewer response is good and satisfies my concerns. With one exception: the authors state that NucHMM is the "first computational tool currently available that is able to genome-wide annotate functional nucleosome states", but "functional nucleosome states" are not precisely defined. More importantly, this discussion must appear in the paper.

2) As far as I can tell, the discussions of related work in the reviewer response does not appear anywhere in the paper.

Otherwise, my concerns are addressed, and the paper is much improved.

**Authors Response**

**Point-by-point responses to the reviewers’ comments:**

***Response to Reviewer #1***

**Major comments:**

*1. In the TNS matrix, the ChIP-seq enrichment signal for a TF is aggregated across genes labelled as {U, M, L, H} and grouped by the nucleosomal states learned by NucHMM. The chromatin-opening ability for a TF is calculated by taking the ratio of its enrichment signal lying within state NucS9 versus lying within states NucS4 and NucS5. Given that no motif enrichment analysis is performed, it is hard to explain using only this ratio whether a TF actually has high chromatin-opening ability. For example, the computed ratio may be high only because the TF’s motif is often present within NucS9 segments of chosen genes but absent in NucS4 and NucS5 segments. This analysis needs to account for whether potential binding sites even exist, perhaps by retaining only those segments that have TF motifs when calculating pioneer capacity. Methods like FIMO can be used to first obtain a set of TF binding sites.*

**Response:** Thanks for your great suggestion. We updated the method of calculating the pioneer capacity with the TF motifs filtering process. As suggested, we firstly used FIMO to filter out all 188 TFs’ peaks by their corresponding TF motifs (note by ${Peaks}_{mtf-filter}$), which were obtained either from JASPAR database or through *de novo* motifs discovery by STREME. We then built the TNS matrix based on ${Peaks}_{mtf-filter}$(the filtered peak results). The following bar plots showed the filtering results (**Suppl. Fig. S17**). As the TR matrix was designed to capture the TF-TF affinity, we kept using the raw TFs peaks as the input to construct the TR matrix. Finally, we only output the pioneer capacity of the TFs that have the motif records in the TF2DNA database. The updated results were shown in **Fig. 5C**, **Suppl. Figs. S19-21**.

*2. Eqn 10: Why is chromatin-opening ability reweighted again when the values are normalized in eqn 9?*

**Response:** As written in the method, the reason why we reweighted the chromatin-opening ability is to avoid a scenario that the large value of ${CO}_{T_{j}}^{'}$ comes from the division of a very small numerator and a smaller denominator. For example, if the numerator is 0.1 and the denominator is 0.001, the ${CO}_{T_{j}}^{'} =100$. Although the ratio is large, the TF is actually lowly enriched in the chromatin-opening region. Thus, it is necessary to reweight the chromatin-opening ability again to diminish this effect.

*3. Eqns 12-13 are still very hard to understand.*

**Response**: Thanks for pointing it out. We rewrote Eqs 11-13 to further clarify the underlying meaning of the equations and provided more explanations (Pages 18-19).

**Minor comments:**

*4. Fig S19: This is a plot of averaged signal and not Fréchet distance. Fréchet distance is only shown for a small segment. Caption needs to be modified*

**Response:** We have modified the Caption as suggested.

***---------------------------------------------***

***Response to Reviewer #2***

**Major comments:**

*1. the authors state that NucHMM is the "first computational tool currently available that is able to genome-wide annotate functional nucleosome states", but "functional nucleosome states" are not precisely defined. More importantly, this discussion must appear in the paper.*

**Response:** Thanks for the suggestion**.** In the introduction section, we defined the functional nucleosome states with the following description: “states encoding combinatorial histone marks and nucleosome organization features that perform specific functions and respond to the different environment and intercellular signaling” (Page 4). We also have rewritten the novelty part in the **Discussion** (Pages 10-11).

*2. As far as I can tell, the discussions of related work in the reviewer response does not appear anywhere in the paper.*

**Response**: We added more discussion of related work in the **Discussion** in the main text as well as in **Additional file1: Suppl. Notes and Figs**.

**Fourth round of review**

**Reviewer 1:**

The authors have now incorporated TF motif analysis to filter ChIP-seq peaks as part of their pioneer capacity analysis. They have also further improved their explanation of how “pioneer capacity” is calculated. However, some of the heuristics described in this calculation are quite unintuitive and raise many questions about the validity of the resulting values. The rest of the paper about the HMM construction and splicing potentiality are interesting and credible, but I remain concerned that I cannot justify the authors’ claims about the pioneer capacity of various TFs on the basis of an opaque analysis. Some specific concerns are below, but at this point I think the authors should consider removing all claims about pioneer capacity and focus on the other results in the paper.

Major comments:

1. The authors describe in Eq. 9 that the chromatin opening ability of a TF is computed as the ratio of enrichment for the given TF in widely spaced nucleosomes (state NucS9) and compact nucleosomes (states NucS4 and NucS5). While this seems reasonable, it is hard to understand why the dynamic change measurement (Eq. 14) considers the change in TF occupancy within the same nucleosome state for different levels of gene expression. Moreover, why is Eq. 14 using chromatin opening ability and not the normalized TF counts used in Eq. 9? Also, why is NucS4 missing from the calculation in Eq. 14?

2. It is hard to understand the reasoning behind Eq. 12 and how it fits in with the authors’ description of pioneer capacity for TFs. The authors claim that if a TF is associated with many other TFs near unexpressed genes then it has a “stronger ability to recruit TFs”. That claim doesn’t seem justified. Also, why is this equation only considering unexpressed genes? Additionally, how is the value RETjTk calculated? Is it taking all nucleosomal states into account or only a subset?

3. In Eq. 11, why is the pioneer ability for a TF the ratio of RTjTk in highly expressed genes and lowly expressed genes? It contradicts the idea presented in Eq. 14 about dynamic change measurement. Likewise, why is the third quantile chosen? What is the overall distribution?

Minor comments:

4. Fig. S19: Which equation does the bar plot describe?

5. Why are the raw peaks used for the construction of the TR matrix?

**Point-by-point responses to the reviewers’ comments:**

***Response to Reviewer #1***

*The authors have now incorporated TF motif analysis to filter ChIP-seq peaks as part of their pioneer capacity analysis. They have also further improved their explanation of how “pioneer capacity” is calculated. However, some of the heuristics described in this calculation are quite unintuitive and raise many questions about the validity of the resulting values. The rest of the paper about the HMM construction and splicing potentiality are interesting and credible, but I remain concerned that I cannot justify the authors’ claims about the pioneer capacity of various TFs on the basis of an opaque analysis. Some specific concerns are below, but at this point I think the authors should consider removing all claims about pioneer capacity and focus on the other results in the paper.*

**Response:** We deeply appreciate your insightful review that you pointed out the interesting and credible results of HMM construction and splicing potentiality in this study. We have further took your advice and now removed all claims about pioneer capacity measure in the manuscript. As such, we have left the remaining comments unaddressed and will incorporate them into our future studies.
